# GRP78/BiP alleviates oxLDL-induced hepatotoxicity in familial hypercholesterolemia caused by missense variants of LDLR in a HepG2 cellular model

**DOI:** 10.1186/s12944-023-01835-x

**Published:** 2023-05-29

**Authors:** Divya Saro Varghese, Deepu Oommen, Anne John, Bassam R. Ali

**Affiliations:** 1 Department of Genetics and Genomics, College of Medicine and Health Sciences, United Arab Emirates University, Al-Ain, United Arab Emirates; 2 Present Address: Indian Institute of Science, C V Raman Road, 560012 Bangalore, India; 3 Zayed Centre for Health Sciences, United Arab Emirates University, Al Ain, United Arab Emirates

**Keywords:** ER stress, oxLDL, ER stress sensors, Hepatotoxicity, Apoptosis

## Abstract

**Background and aims:**

The accumulation of misfolded proteins, encoded by genetic variants of functional genes leads to Endoplasmic Reticulum (ER) stress, which is a critical consequence in human disorders such as familial hypercholesterolemia, cardiovascular and hepatic diseases. In addition to the identification of ER stress as a contributing factor to pathogenicity, extensive studies on the role of oxidized Low-Density Lipoprotein (oxLDL) and its ill effects in expediting cardiovascular diseases and other metabolic comorbidities are well documented. However, the current understanding of its role in hepatic insults needs to be revised. This study elucidates the molecular mechanisms underlying the progression of oxLDL and ER stress-induced cytotoxicity in HepG2.

**Methods:**

HepG2 cells stably expressing wild-type Low-Density lipoprotein receptor (WT-LDLR) and missense variants of LDLR that are pathogenically associated with familial hypercholesterolemia were used as the in vitro models. The relative mRNA expression and protein profiles of ER stress sensors, inflammatory and apoptotic markers, together with cytotoxic assays and measurement of mitochondrial membrane potential, were carried out in HepG2 cells treated with 100 µg per ml oxLDL for 24 to 48 h. 1-way or 2-way ANOVA was used for statistical analyses of datasets.

**Results:**

ER stress responses are elicited along all three arms of the unfolded protein response (UPR), with adverse cytotoxic and inflammatory responses in oxLDL-treated conditions.

Interestingly, oxLDL-treated ER-stressed HepG2 cells manifested intriguingly low expression of BiP- the master regulator of ER stress, as observed earlier by various researchers in liver biopsies of Non-Alcoholic Steatohepatitis (NASH) patients. This study shows that overexpression of BiP rescues hepatic cells from cytotoxic and inflammatory mechanisms instigated by ER stress in combination with oxLDL, along the ER and mitochondrial membrane and restores cellular homeostasis.

**Conclusion:**

The data provide interesting leads that identify patients with familial hypercholesterolemia conditions and potentially other Endoplasmic Reticulum Associated Degradation (ERAD) diseases as highly susceptible to developing hepatic insults with molecular signatures like those manifested in Non-Alcoholic Fatty Liver Disease (NAFLD) and NASH.

**Limitations and future perspectives:**

Although the use of HepG2 cells as the model is a major caveat of the study, the findings of this research may be used as the pilot study to expand further investigations in primary hepatocytes or iPSC- derived cellular models.

**Supplementary Information:**

The online version contains supplementary material available at 10.1186/s12944-023-01835-x.

## Introduction

Intracellular lipid homeostasis is maintained by multiple mechanisms that tightly regulate lipids' uptake, storage, and clearance. Abnormal concentrations of lipids such as cholesterol, low-density lipoprotein cholesterol (LDL-c), and triglycerides are manifested in dyslipidemic conditions. Familial hypercholesterolemia (FH) is a genetic disorder that is mainly inherited in an autosomal dominant manner and is characterized by elevated plasma LDL-c levels that can lead to premature atherosclerotic cardiovascular disease (ASCVD) and early death [1]. Approximately 60–80% of FH cases are attributed to inherited mutations in LDLR (Low-density lipoprotein receptor), of which about 46% are accounted for by missense variants of LDLR [2]. A significant proportion of FH-causing LDLR variants lead to the retention of the mutant receptor in the ER via the ER- associate protein degradation (ERAD)[3]. ERAD has been implicated in mechanisms of numerous human diseases, including FH [4–8].

In FH conditions, reduced clearance of serum LDL-c due to inefficient LDL uptake by LDLR increases the residence time of LDL in circulation and favors its oxidation [9, 10]. The oxidized derivative of Low-Density Lipoprotein (oxidized LDL or oxLDL), a by-product of oxidative stress, is a pathogenic hallmark of lipid dysfunction-associated metabolic diseases, which eventually lead to comorbidities affecting the cardiovascular system, liver, pancreas, and kidney [11, 12]. Growing evidence connects oxidized LDL and ER stress, which effectuates atheroprogression and other cardiovascular diseases [13, 14]. Being the central regulator of lipid homeostasis, the liver plays a significant role in the clearance of plasma lipids, which is mediated by LDLR. The endoplasmic reticulum (ER) is instrumental in sensing and regulating lipid homeostasis and protein homeostasis [15] simultaneously. Hepatocytes are structurally rich in ER, the major cellular hub of protein synthesis, thus making the hepatocytes more sensitive to the disruption of normal ER function. When ER homeostasis is perturbed, sensor proteins on ER membranes initiate Unfolded protein response (UPR). Physiologically, the ER's Unfolded Protein Response (UPR) is essential for maintaining cellular homeostasis in hepatocytes during metabolism and protein secretion. However, UPR signaling also drives the pathogenesis of liver disease through its involvement in inflammatory responses, steatosis, hepatocyte apoptosis, and fibrosis, highlighting the importance of the ER in physiological hepatocyte processes. In addition to apoptosis, the cross talk between multiple routine cell death mechanisms such as autophagy [15, 16], necrosis [17, 18], necroptosis [19–21], ferroptosis [22–24], and pyroptosis [25–27] classified under the umbrella-term PANoptosis, contribute to hepatotoxicity and liver injury. It is important to note that the intricate cross talk between these mechanisms with overlapping signaling pathways magnify the challenges in identifying targets for therapy, mainly due to the discrepancies in the perplexing findings.

LDLR plays a pivotal role in maintaining cholesterol homeostasis in hepatic cells. In spite of accumulating data on hepatic insults that coincide with excess LDL-cholesterol as in hypercholesterolemia, the cumulative effect of ER stress and oxLDL- induced stress in liver dysfunction is still less-explored [28–30]. Previously, members of this group had identified the defective trafficking and proteasomal degradation of ER-retained, missense variants of LDLR (p.D482H-LDLR and p.C667F-LDLR) that are pathogenically associated with FH, in HeLa and HEK293T cells [31]. This prompted us to investigate the molecular mechanisms of hepatotoxicity caused by the jeopardizing combination of oxLDL and ER stress.

However, contrary to the expected upregulation of the master regulator BiP/GRP78 under conditions of ER stress, as in the previous reports from this group and other researchers, this study reports the downregulation of BiP/GRP78 in HepG2 cells stably expressing WT- or missense variants of LDLR. This data provides evidence for the downregulation of BiP in hepatic tissues exhibiting FH conditions, similar to the depleted BiP expression manifested in the hepatic tissues of NASH patients. Further, the protective function(s) of GRP78/BiP in alleviating ER stress and rescuing hepatic cells from being destined to cell death that culminates in disrupted liver function and associated diseases was investigated.

## Materials and methods

### Generation of pathogenic ER retained missense variants of LDLR

All experiments in this manuscript refer to the ORF of the human LDLR clone with Accession Number NM_000527. The C-terminal Myc-DDK tagged LDL Receptor in the pCMV6 vector was purchased from Origene. (Cat No. RC200006). To generate the two missense, site-directed Mutagenesis of LDLR -Myc-DDK plasmid was carried out using High Fidelity Pfu Ultra Polymerase (Agilent Technologies, Cat. No. 600382) variants of LDLR- p.D482H and p.C667F, that are pathogenic in FH patients. Primers used to introduce mutations at G > C, C > T for p.D482H and p.C667F at G > T were designed by PrimerX software (PrimerX (bioinformatics.org). Direct sequencing of plasmids was carried out on the ABI 3130xl genetic analyzer (Applied Biosystems, Waltham, MA, USA) to confirm the mutations at the respective positions. Table 1 enlists all the primers used in the study. For the generation of stably transfected HepG2 cell lines by lentiviral transduction, pLenti C-Myc-DDK-P2A Puro Lentiviral empty vector (Origene, Cat. No. PS100092), wild type-, D482H-, and C667F-LDLR plasmids were sequentially digested with MluI (NEB, Cat. No. R0198S) and AsiS/SgfI (NEB, Cat. No. R0630S). The ORF of wild-type and missense variants of LDLR was ligated into pLenti C-Myc-DDK-P2A Puro Lentiviral empty vector (Origene, Cat. No. PS100092), treated with Antarctic phosphatase (NEB, Cat No. M0289S), using T4DNA ligase (NEB, Cat. No. M020L). The ligated products were transformed into Stbl3-competent cells and selected using chloramphenicol. Plasmids isolated from positive clones were isolated and used for viral transduction to generate stable lines.

**Table 1:** List of all the primers used in the study

Sl. No	Primer Name	Primer sequence 5`-3`
1	***XBP1-s*** **-For**	**GCAGGTGCAGGCCCAGT**
2	***XBP1-s*** **- Rev**	**GAATGCCCAACAGGATATCAGACT**
3	***P58IPK*** **-For**	**GGTTCGGTATTCCCCTTCTTCCT**
4	***P58IPK-*** **Rev**	**AGTAGCCCTCCGATAATAAGCAA**
5	***EDEM1-*** **For**	**CGGACGAGTACGAGAAGCG**
6	***EDEM1-*** **Rev**	**CGTAGCCAAAGACGAACATGC**
7	***CHOP-*** **For**	**GGAGAACCAGGAAACGGAAAC**
8	***CHOP-*** **Rev**	**TCTCCTTCATGCGCTGCTTT**
9	***BiP-*** **For**	**CATGGTTCTCACTAAAATGAA**
10	***BiP-*** **Rev**	**GCTGGTACAGTAACAACTG**
11	***ATF4-*** **For**	**GGTCAGTCCCTCCAACAACA**
12	***ATF4-*** **Rev**	**CTATACCCAACAGGGCATCC**
13	***ATF6-*** **For**	**AATCCGCTTGTCAGTCTCGC**
14	***ATF6-*** **Rev**	**GCCTCTGGTTCTCTGACACA**
*15*	***PERK-*** **For**	**TCATCCAGCCTTAGCAAACC**
16	***PERK-*** **Rev**	**ATGCTTTCACGGTCTTGGTC**
17	***GAPDH-*** **For**	**AAGGTGAAGGTCGGAGTCAA**
18	***GAPDH-*** **Rev**	**AATGAAGGGGTCATTGATGG**
19	***Sgfl-*** ** FL-WT** ***-LDLR-*** ** F1**	**GAGGCGATCGCCATGGGGCCCTGG**
20	***MluI-*** **FL-WT** ***-LDLR-*** **R2580**	**GCGTACGCGTCGCCACGTCATCCTC**
21	***LDLR-*** **Seq-R377**	**AACTGCCGAGAGATGCACTT**
22	**LDLR-SDM-D482H-For**	**CGACGGGCTGGCTGTGCATTGGATCCACAGCAACAT**
23	**LDLR-SDM-D482H-Rev**	**ATGTTGCTGTGGATCCAATGCACAGCCAGCCCGTCG**
24	**LDLR-SDM-C667-For**	**GAGGAGTGAACTGGTTTGAGAGGACCACCC**
25	**LDLR-SDM-C667-Rev**	**GGGTGGTCCTCTCAAACCAGTTCACTCCTC**
26	**V2 -Lenti-For**	**AGAGCTCGTTTAGTGAA**
27	**LR50-Lenti-Rev**	**CAGAGGTTGATTATCGATAAG**

### Animal cell culture and lentiviral transduction to generate stable HepG2 cells

Hepatocellular carcinoma cell line, HepG2 (ATCC HB-8065), were maintained in RPMI 1640 Media containing Glutamax (Gibco, Cat. No. 61870036), supplemented with 10% Heat inactivated fetal bovine serum (Gibco, 10,500–064) and 100 U per ml of penicillin–streptomycin solution (Gibco, Cat. No. 15140–122) in a humidified incubator at 37 °C supplied with 5% CO_2_.

Pseudoviral particles expressing the wild-type and missense variants of LDLR were generated using HEK293T cells using the Lentiviral Packaging Kit as per the manufacturer’s instructions (Origene, Cat. No. TR3007). In brief, HEK293T cells maintained in antibiotic-free media were co-transfected with 5 µg of FLAG-tagged empty p-Lenti vector or ORF of WT-/D482H-/C667F-LDLR expression constructs and 6 µg of packaging plasmid diluted in 1.5 ml OptiMEM (Gibco, Cat. No. 31985062). In addition, 33 µl of Turbofectin transfection reagent (Origene, Cat. No. TF81002) was mixed with the diluted DNA mix. 72 h post-transfection, the supernatant containing viral particles was centrifuged at 3000 rpm (3,300xg) for 5 min, filtered using a 0.45 µm cell strainer (Corning, Cat No.352340), and stored for further use. Polybrene (Millipore, Cat. No. TR-1003-G) was used at 8 µg per ml as the transducing reagent to infect HepG2 cells with p-Lentiviral particles carrying the FLAG tag or FLAG-tagged ORFs of WT/variants of LDLR. Approximately, > 95% transduction efficiency and minimal cell death were obtained with a seeding density of 3 × 10^4^ cells infected with viral particles at a multiplicity of Infection (MOI) of 3. The transduced stable cells were maintained media at a final concentration of 10 µg per ml puromycin for 14 days to select the positive clones.

### Electroporation and oxidized LDL treatment

HepG2 cells were electroporated with GRP78/BiP plasmid (Origene, Cat. No. SC108086) using the Neon transfection system (Thermo Fisher Scientific, Cat. No. MPK5000) with slight modifications to the manufacturer’s instructions. In brief, 1 × 10^6^ cells and 200–400 ng plasmid DNA were nucleofected with 3 pulses of 1,100 V at a pulse width of 40 ms. 48 h post electroporation, HepG2 cells were treated with commercially available oxidized LDL (Thermo Fisher Scientific, Cat. No. L34357) at 100 µg per ml growth media for 24 to 48 h. Tunicamycin at a concentration of 2 µg per ml (Sigma, Cat. No. T7765) was used as the inducer of ER stress response and as the positive control in the experiments. DMSO was used as the vehicle control, at a volume of 0.4 µl per ml culture media, similar to the volume of tunicamycin used in all experiments. Cells were harvested at specific time points (8, 12, 16, 24, and 48 h) post-treatment and lysed for further assays.

### Immunofluorescence and confocal microscopy

For immunofluorescence, stable HepG2 cells expressing FLAG-tagged wild-type or missense variants of LDLR were grown on sterile coverslips. 24 h post electroporation, the cells were fixed with methanol for 5 min at -20 °C, washed with PBS, and blocked with 1% BSA in PBS overnight at 4 °C. The cells were incubated for 1 h at room temperature in primary antibodies diluted in blocker solution. The cells were washed several times in PBS and incubated in secondary antibody at room temperature in dark for 45 min. The antibodies used, and their dilutions are as follows- mouse monoclonal anti-FLAG antibody, 1 in 400 in 1%BSA (Sigma, Cat. No. A1804), rabbit polyclonal anti-Calnexin antibody, 1 in 50 in 1%BSA (Cell Signaling Technology, Cat. No. 2369S), rabbit polyclonal anti-Na^+^K^+^ ATPase antibody, 1 in 50 in 1% BSA (Santa Cruz, Cat. No, sc-2880), Alexa 568 conjugated donkey anti-mouse antibody (Invitrogen, Cat. No. A10037), and Alexa 488 conjugated goat anti-rabbit antibody (Cell Signaling Technology, Cat. No. 4412S). The coverslips were air dried and mounted using a fluorescence mounting medium (DAKO, Cat. No. S3023). Images were captured using the 100-X oil immersion objective of a Nikon Eclipse confocal microscope equipped with FITC and TRITC filters.

### RNA extraction, cDNA synthesis, and Real-Time PCR

Cells were harvested, and total RNA was isolated using the PureLink™ RNA Mini Kit (Thermo Fisher Scientific, Cat. No. 12183020) as per the manufacturer’s instructions. First-strand cDNA synthesis of 250–500 ng total RNA was done using GoScript Reverse Transcriptase System (Promega, Catalog No. A5004). Relative mRNA expression of target genes was checked using SYBR® Green Real-Time qPCR Master Mix (Thermo Fisher Scientific, Cat. No. 4309155) in QuantStudio™ 7 Flex Real-Time PCR System, 96-well block (Applied Biosystems™, Cat. No. 4485701). The primer sequences used are listed in Table 1.

### Protein extraction, SDS-PAGE, and Western blotting

At the specific time points, treated and non-treated cells were washed in ice-cold PBS before harvesting. Total protein was extracted in 1X RIPA buffer (Cell Signaling Technology, Cat. No. 9806) containing Halt™ protease and phosphatase inhibitor cocktail (Thermo Fisher Scientific, Cat. No. 78441), and the crude lysate was centrifuged at 14,000 rpm (14,400xg) for 20 min at 4 °C. The supernatant containing the total protein lysate was quantified using BCA Protein Assay Kit (Thermo Fisher Scientific, Cat. No. 23225), and optical density was measured at 490 nm using the TECAN Infinite M200 Pro plate reader. 35–40 µg protein was loaded on 4–12% gradient Tris-MOPS-SDS PAGE gels (GenScript Express plus PAGE Gels, Cat. Nos. M41210 and M41215) or 6% Tris–Glycine SDS-PAGE gels. After electrophoresis, the gels were immunoblotted onto PVDF membranes (Thermo Fisher Scientific, Cat. No. 88518). The membranes were blocked in 5% non-fat milk or Bovine Serum Albumin (Sigma, Cat. No. A2153) for 1 h at room temperature and incubated overnight in the primary antibody at 4 °C. The membranes were washed 4 times in TBS and incubated in HRP-conjugated secondary antibody for 1 h at room temperature. The bands were visualized by Super Signal West Pico chemiluminescent substrates as per the manufacturer’s instructions (Thermo Fisher Scientific, Cat. Nos. 34080) in a Sapphire Biomolecular Imager, Azure Biosystems. The antibodies used and their dilutions are as follows: Rabbit polyclonal anti-Alpha Tubulin (1 in 1000, Cell Signaling Technology, Cat. No. 2144S), Rabbit polyclonal anti-GAPDH (1 in 1000, Cell Signaling Technology, Cat. No. 2118S), Rabbit polyclonal anti-FLAG (1 in 1000, Cell Signaling Technology, Cat. No. 2368S), Rabbit polyclonal anti-IRE1A (1 in 500, Cell Signaling Technology, Cat. No. 3214S), Rabbit polyclonal phospho-IRE1A (Ser724) (1 in 500, Novus Biologicals, NB100-2323), Rabbit polyclonal anti-eIF2A (1 in 500, Cell Signaling Technology, Cat. No. 9722S), Rabbit polyclonal anti phospho-eIF2A (Ser51) (1 in 500, Cell Signaling Technology, Cat. No. 9721S), Rabbit polyclonal anti-XBP1 Antibody (1 in 1000, Novus Biologicals, NB2-20,917), Rabbit polyclonal anti-GRP78/BiP (1 in 1000, Cell Signaling Technology, Cat. No. 3177S), Rabbit polyclonal anti-BIM (1 in 300, Novus Biologicals, NBP2-67,456), Rabbit polyclonal anti-phospho BIM (1 in 300, Cell Signaling Technology, Cat. No. 12433S), Mouse polyclonal anti-CHOP (1 in 250, Cell Signaling Technology, Cat. No. 2895S), Rabbit polyclonal total Caspase 3 antibody (1 in 1000, Cell Signaling Technology, Cat. No. 9662S), Rabbit polyclonal anti-cleaved caspase 3 (1 in 250, Cell Signaling Technology, Cat. No. 9664S), Mouse monoclonal anti-JNK (1 in 500, Cell Signaling Technology, Cat. No. 3708), Rabbit polyclonal anti-phospho JNK (Thr183/Tyr 185) (1 in 250, Cell Signaling Technology, Cat. No.9251), Mouse monoclonal anti-Cytochrome *c* antibody (1 in 300, Thermo Fisher Scientific, Cat. No. MA5-11,674), Goat-raised, HRP-conjugated secondary antibodies against Mouse (1 in 30,000, Cat. No. 115–035-1661) and Rabbit (1 in 30,000, Cat. No. 111–035-1441) were purchased from Jacksons ImmunoResearch Europe LTD.

Blots were quantitatively analysed by densitometric analysis of respective signal intensities normalized to Alpha tubulin or GAPDH as loading controls. Representative images are presented in each panel. Densitometric analysis was carried out using the QuickFigures plugin of ImageJ software https://doi.org/10.1371/journal.pone.0240280. Due to the large number of targets [12] and samples [20] tested by Western blotting, membranes were cropped before antibody incubation as described. After capturing the signals from blots immunoprobed with phospho-antibodies, the membranes were stripped and re-probed with the respective total protein antibodies. For each cell type studied, the protein lysates of respective controls and treatments were loaded on the same gel and incubated in respective antibodies by cropping the membranes such that molecular weight markers above and below the band(s) of interest are included. Electrophoresis and Western Blotting were repeated with the same lysates to detect protein targets with overlapping molecular weights, ensuring that the respective controls and test samples were separated on the same gel and blotted onto the same membrane. Comparisons were carried out between non- treated controls, expressing endogenous target proteins, and treated tests that were manipulated with empty vector or LDLR constructs, on the same membrane. These comparisons were made to rule out any biases that may arise due to the genetic manipulation introduced. For convenient representation of expression profiles of the targets in each cell type, cropped membranes demarcating the site of splicing are represented in the figures. Alpha tubulin or GAPDH presented in each panel is a representative image. GraphPad Prism 6 software was used to generate bar graphs for the total protein or housekeeping control-normalised blots and represented on the same graph to compare the expression profile. Image panels were created using Adobe Illustrator CS6.

### Cytotoxicity assay-Lactate Dehydrogenase release

After 48 h of treatment, Lactate Dehydrogenase (LDH) released into the media was measured using the CytoTox 96® non-radioactive cytotoxicity assay kit (Promega, Cat. No. G1780) according to the manufacturer’s instructions. Absorbance was measured at 490 nm using the TECAN Infinite M200 Proplate reader.

### FACS-based measurement of mitochondrial membrane potential

Flow cytometry-based assay was carried out to measure the mitochondrial membrane potential (ΔΨm) using the MitoProbe™ JC-1 Assay Kit (Thermo Fisher Scientific, Cat. No. M34152) as per the guidelines mentioned in the kit. JC-1 dye enters the mitochondria and forms aggregates that emit red fluorescence at 590 nm. JC-1 monomers emit green fluorescence, and hence an increase in green fluorescence indicates a disrupted membrane potential caused by a leaky mitochondrial membrane. In brief, 48 h after treatment, cells were trypsinized and pelleted at 1000 rpm for 5 min. The cell pellet was homogenously resuspended in PBS containing the cationic dye, JC-1 (5’,6,6’-tetrachloro-1,1’,3,3’-tetraethylbenzimidazolylcarbocyanine iodide), to obtain a final concentration of 4 µM. JC-1stained cells were incubated at 37 °C for 30 min in a CO_2_ incubator. For the positive control, before JC-1 staining, the cell suspension was preincubated in PBS containing 50 µM CCCP (carbonyl cyanide 3-chlorophenylhydrazone), a mitochondrial membrane potential disruptor, for 15 min at 37 °C in a CO_2_ incubator. CCCP/JC-1-stained cells were washed in PBS, and 1 × 10^4^ cells were analyzed on a FACS CANTO II Flow cytometer using BD FACS Diva™ Software v6.x. Non-stained control cells were used for gating and the P1 population of cells was placed at the center of the FSC-SSC plot. The PMT voltage of the gated P1 population of non-stained control cells was adjusted to emit ‘zero fluorescence’ when excited at the respective wavelengths of the FITC and PI lasers. These parameters were fixed for the corresponding treated or stained cells. In the dot plot representing the cell population distributed in either of the four quadrants (Q1-Q4), the Y-axis corresponds to the JC-1 aggregates that emit red fluorescence, and the X-axis corresponds to the JC-1 monomers that emit green fluorescence. The ratio of cells in the Q2 and Q4 populations was used to assess the mitochondrial membrane potential status in treated versus non-treated cells.

### Statistical analysis

The number of biological replicates is mentioned in the corresponding figure legends, and the bar diagrams represent the mean ± standard deviation (SD). The images presented are representative images for each target evaluated. In experiments carried out under non-treated conditions, statistical analyses were done using GraphPad Prism6 software (1-way ANOVA using Dunnett’s test or 2-way ANOVA using Turkey’s post hoc test), as mentioned in each figure legend. Results were analysed based on comparisons between non-electroporated HepG2 cells or cells stably expressing empty vector as control versus stable HepG2 expressing FLAG-tagged WT/variants of LDLR. For oxLDL treatment regimes, the respective non-treated cells were used as ‘control’ and the treated cells as ‘test’ to compare the expression of each target protein studied. The asterisk (*) or hashtag (#) represent differences being significant (* or # *P* < 0.05, ** P < 0.01, *** *P* < 0.001, *P* < 0.0001).

## Results

### Generation of wild-type and ER retained missense variants of LDLR by site-directed mutagenesis and stable lentiviral transduction of HepG2 cells

**Fig. 1:**
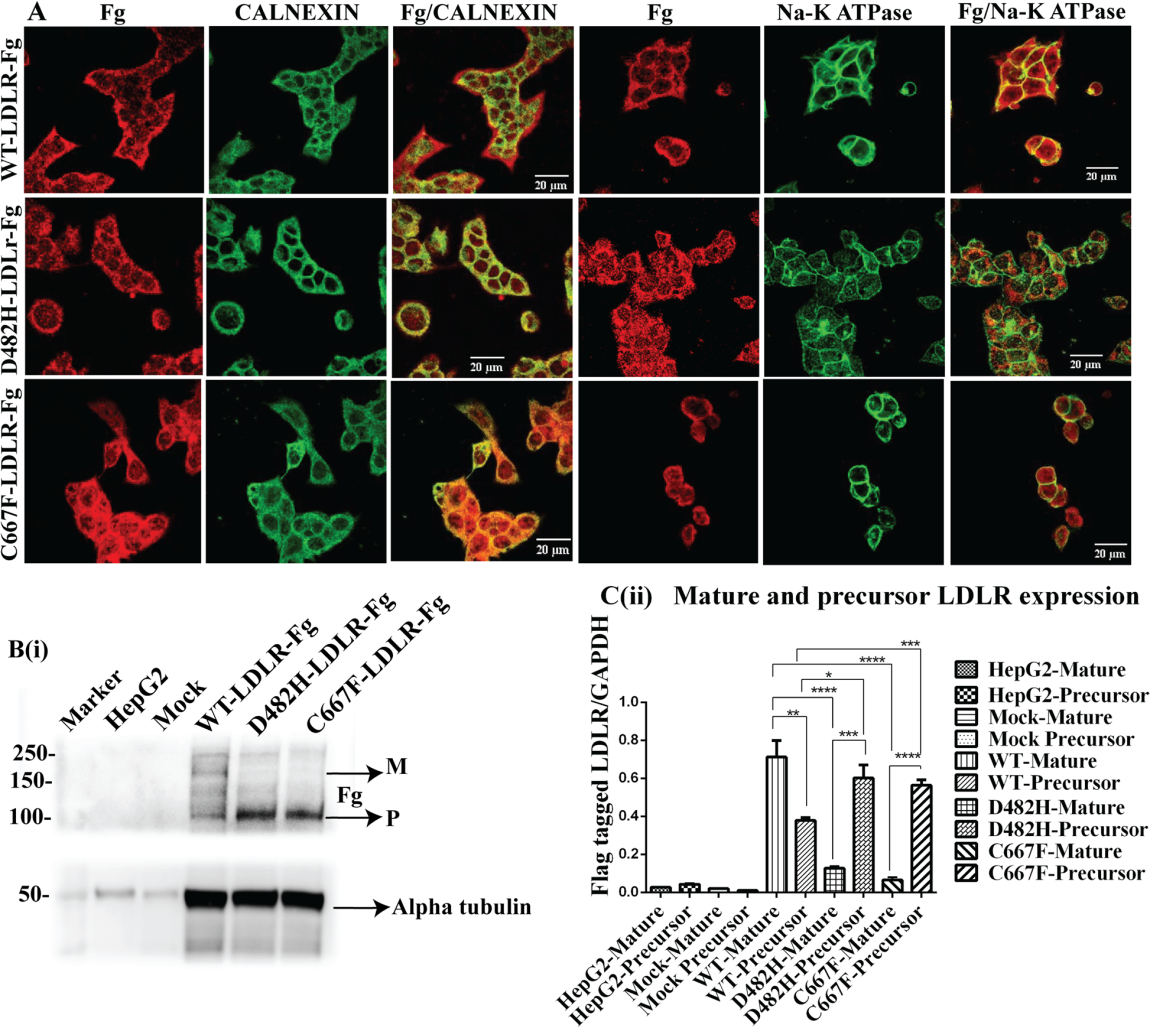
**A-B** Generation of lentiviral mediated stable HepG2 lines expressing Wild Type/missense variants of LDLR. **A** Immunofluorescence with anti-FLAG (Fg), anti-Calnexin (CNX), and *anti*- Na^+^K^+^ ATPase antibodies to understand the subcellular localization of FLAG-tagged (Fg) WT-LDLR, D482H-LDLR, and C667F-LDLR in stable HepG2 cells. Wild-type LDLR is expressed predominantly on the plasma membrane, whereas D482H-LDLR and C667F-LDLR are retained in the Endoplasmic Reticulum (ER). Calnexin (CNX) was used as the ER marker and Na^+^K^+^ ATPase as the marker of the plasma membrane. The color images represent merged images of red (Fg) and green (Calnexin or Na^+^K^+^ ATPase) channels. The fluorescent images were captured using the 100-X oil immersion objective of a Nikon Eclipse 2000 Confocal Microscope and color-enhanced using ImageJ software. Scale bar = 20 µm. **B** (i)) Western blotting with anti-FLAG antibody confirms the expression of mature (M) and precursor (P) LDLR protein of 160 kDa and 120 kDa, respectively in WT-LDLR expressing HepG2. The missense variants D482H-LDLR and C667F-LDLR fail to express the mature 160 kDa protein and confirm the ER retention by the prominent expression of the immature 120 kDa protein. The FLAG tag of 25 amino acids is not expressed in Non-transduced HepG2 control cells and not detected in mock transduced HepG2. Alpha tubulin represents the loading control. (**B**(ii)). The fold change of Alpha tubulin normalized mature and immature forms of FLAG-tagged LDLR is represented as mean + SD. *n* = 3. Unpaired students’ *t*-test, two-tailed, *P* < 0.05 = *, *P* < 0.01 = **

The missense variants of LDLR- p.D482H and p.C667F were generated by site-directed mutagenesis of FLAG-tagged Wild Type (WT) LDLR plasmid DNA and subsequently sequenced using Sanger’s Direct DNA Sequencing to confirm the successful introduction of nucleotide substitutions (Supplemental Fig. 1). HepG2 cells were stably transduced with the generated plasmids using lentiviruses. Hereafter, stable HepG2 cells expressing FLAG-tagged empty vector, WT-LDLR, and the missense variants of LDLR (p.D482H and p.C667F) are denoted as HepG2^mock^, HepG2^WT+^, HepG2^D482H+^, and HepG2^C667F+^ in the manuscript. The cellular localization of these plasmids in cells stably expressing WT (HepG2^WT+^) and variants (HepG2^D482H+^ and HepG2^C667F+^) as well as the efficiency of transduction was confirmed by immunostaining with FLAG-tagged antibody (Fig. 1A). WT-LDLR was expressed predominantly on the plasma membrane of HepG2^WT+^, colocalizing with the plasma membrane marker- Na^+^K^+^ ATPase, and in the ER, along with the ER-resident chaperone- Calnexin. In HepG2^D482H+^ and HepG2^C667F+^, LDLR failed to reach the plasma membrane and is retained in the ER, co-expressing with Calnexin. The partially glycosylated precursor form of LDLR is synthesized in the ER. This immature protein transits from the ER to the Golgi complex, where it gets fully glycosylated to the mature protein and gets translocated to the plasma membrane to perform its functions as the receptor of LDL. To elucidate the expression pattern of LDLR in HepG2^WT+^, HepG2^D482H+^, and HepG2^C667F+^, a western blot was performed by probing with anti-FLAG antibody and bands of LDLR precursor (120 kDa) and mature (160 kDa) forms were identified (Fig. 1B(i)). The absence of the slowly migrating band corresponding to the fully glycosylated mature LDLR of 160 kDa in HepG2 cells expressing the missense variants D482H and C667F reiterates the ER retention of these missense variants. The expression of fully glycosylated mature LDLR was significantly reduced in mutants HepG2^D482H+^ and HepG2^C667F+^. Non transduced HepG2 and Mock-transduced HepG2 represent the control cells used for the experiments. The FLAG tag of 25 amino acids is not expressed in the non-transduced lysates and undetected in the mock-transduced control cells (Fig. 1Bi). The differences between WT LDLR expression patterns in HepG2^WT+^ and mutants in HepG2^D482H+^ and HepG2^C667F+^ were significant (Fig. 1B(ii)). Overexpression of wildtype LDLR in HepG2 cells causes excessive protein accumulation in the ER, awaiting to be correctly folded until it exits the ER for further maturation. This explains the expression of immature protein retained in the ER in HepG2^WT+^ cells.

### Activation of UPR in HepG2 cells expressing mutant forms of LDLR

**Fig. 2:**
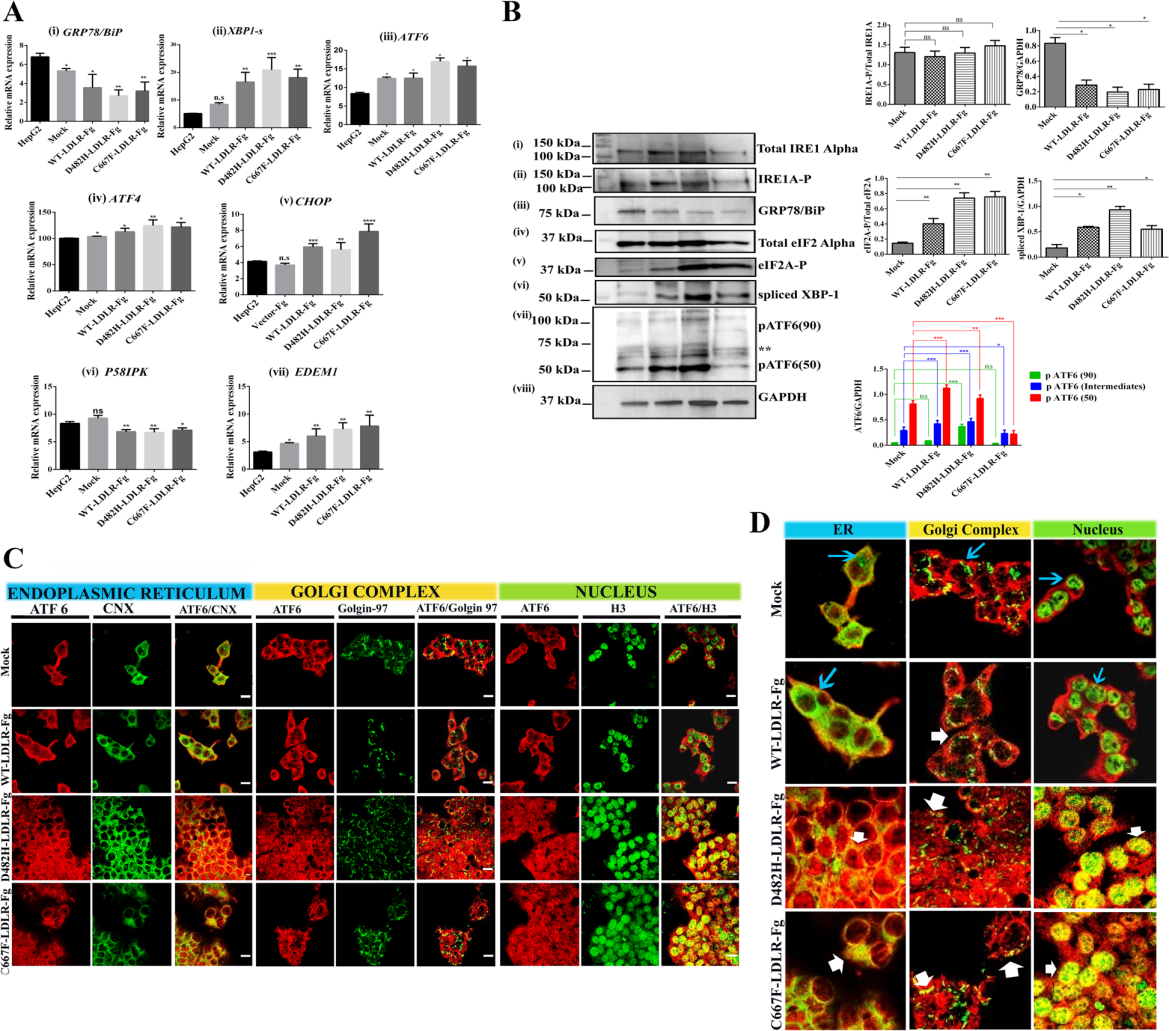
**A**-**D** Upregulation of ER stress response along all three arms of the UPR in HepG2 cells expressing FLAG-tagged-WT/missense variants of LDLR. **A** Relative mRNA expression of the three arms of ER stress sensors and their downstream targets, namely (i) *GRP78/BiP* (ii) *XBP-1(s)* (iii) *ATF6* (iv) *ATF4* (v) *CHOP* (vi) *P58IPK* and (vii) *EDEM1*. Comparisons were done between non-transduced HepG2 and transduced HepG2 and represented as mean + SD. Dunnett’s 1-way ANOVA; p < 0.05, p < 0.01 = **, *n* = 3 replicates. **B** Western blotting with antibodies to (iii) the ER chaperone-BiP, and UPR arms confirm the activation of ER stress response along the (i, ii, and vi) IRE1Alpha/spliced XBP-1-, (iv and v) PERK/eIF2A-, and (vii) ATF6 branches, along with (viii) GAPDH. The inactive, ER- membrane-bound ATF6 protein of 90 kDa is represented as pATF6 (90). pATF6(50) represents the activated and cleaved nuclear fragment, whereas ‘**’ denotes the intermediate isoforms of ATF6 ((pATF6(intermediates)) detected on Western blots. The corresponding histograms represent the relative fold change of phosphorylated to Total protein or GAPDH, the loading control. Data are represented as mean + SD. Statistical analyses were carried out using Students’ unpaired, two-tailed *t*- test or 2-way ANOVA (Turkey’s post hoc), *P* < 0.05 = * or #, *P* < 0.01 = **, *P* < 0.001 = ***, ns = not significant; *n* = 2 replicates. **C** Immunofluorescence image panel illustrating ATF6 translocation from ER to Golgi complex and nucleus in mock, WT-, D482H- and C667F-expressing HepG2 cells. The white horizontal lines at the bottom right corner of each merged image represents the scale bar = 20 µm. **D** Merged panels zoomed to 50% magnification. The fluorescent images were captured using the 100-X oil immersion objective of a Nikon Eclipse 2000 Confocal Microscope and color-enhanced using ImageJ software. Color images were obtained by merging red (ATF6) and green (Calnexin, Golgin-97, or H3) channels. ER marker- Calnexin (CNX), Golgi complex marker- Golgin-97, Nuclear marker- H3 (Histone3). The thin blue arrows in the merged panels are used to highlight regions emitting signals from the green channel alone and represent regions where ATF6 does not express with the respective organelle marker. The thick white arrows in these panels highlight yellow fluorescence indicating overlapping signals from the green and red channels and denote regions where ATF6 co-expresses with the respective organelle markers recorded from both the green

ER retention of p.D482H-LDLR and p.C667F-LDLR triggers ER stress response as demonstrated by upregulated transcripts of spliced X-box protein (*XBP-1 s*), the downstream target of the IRE1A arm of UPR [31]. To investigate whether the ER stress response is initiated by all three arms of the UPR, the relative mRNA expressions of these targets were evaluated using Real-Time PCR (Fig. 2A). In conjunction with previous reports that describe the cellular mechanism of pathogenicity of FH-causing LDLR variants, ER retention of the missense variants induced ER stress and it upregulated the UPR arms [31]. The transcripts of all three UPR arms and selected targets that play a vital role in ER Associated Degradation (ERAD) and ER Quality Control (ERQC) were assessed. Non-transduced HepG2 cells were used as the control for the experiment to evaluate the basal level expression of all the targets studied and to confirm that the changes in transcript profile were not caused by the genetic manipulation employed. Compared to the non-transduced/ HepG2^mock^, *XBP-1(s)*, *ATF4*, *CHOP,* and *ATF6* representing the immediate targets of the three branches of UPR were upregulated in HepG2^WT+^, HepG2^D482H+^, and HepG2^C667F+^, with higher fold change in ER retained cells (Fig. 2A(ii-v)). Interestingly, *P58IPK*- the PERK inhibitor, was downregulated in HepG2^WT+^, HepG2^D482H+^, and HepG2^C667F+^ (Fig. 2A(vi)). In addition to the surge in transcripts of the UPR signal activators, overexpression of WT-, D482H-, and C667F-LDLR resulted in a simultaneous enrichment of *EDEM1* (ER-degradation enhancing α-mannosidase-like Protein-1), suggesting the activation of the cell’s survival mechanisms to combat ER stress by recruiting ERAD components ((Fig. 2A(vii)). In spite of the activation of all three ER sensors and ERAD components, there was a riveting reduction in the transcripts of GRP78/BiP- the ER stress master regulator.

Figure 2B (i-viii) describes the activation of the three ER stress sensors- IRE1A/spliced XBP-1, PERK, and ATF6 assessed by immunoblotting. Comparisons were made as described in the Methods section**.** The corresponding graphs representing GAPDH normalized protein expression depict the fold change in ER-retained variants and indicate the activation of all three ER stress sensor arms- IRE1A/XBP1-s, PERK/eIF2A, and ATF6 in HepG2^WT+^, HepG2^D482H+^, and HepG2^C667F+^ compared to the HepG2^mock^. The retention of the LDLR missense variants did not cause a significant fold change in the expression of phosphorylated IRE1A compared to WT-LDLR/empty vector-expressing cells. However, ER retention of these variants resulted in the activation of the RNase domain of IRE1A, as indicated by an evident five-fold increase in XBP-1(s) protein. Likewise, the activation of the PERK/eIF2A arm was evident from the enhanced expression of phosphorylated eukaryotic Initiation Factor Alpha (eIF2A), with a two-to-four-fold upregulation of phosphorylated eIF2A in HepG2^D482H+^ and HepG2^C667F+^ compared to the HepG2^WT+^ and HepG2^mock^. Proteolytic cleavage of ATF6 was detected in LDLR-WT/variant-expressing cells. In addition to the inactive, glycosylated, or non-glycosylated isoforms of the transmembrane protein of 90 kDa (pATF6-P), the cleaved N-terminal cytosolic domain of ~ 50 kDa (pATF6-N), together with intermediate isoforms of ATF6, as reported by Jin et al*.* [32], were detected in the total protein lysates of HepG2^WT+^, HepG2^D482H+^, and HepG2^C667F+^, with predominant expression of pATF6-N in HepG2^WT+^ and HepG2^D482H+^ than in HepG2^C667F+^.

To confirm the ER-Golgi-nuclear trafficking of activated ATF6, HepG2^mock^, HepG2^WT+^, HepG2^D482H+^, and HepG2^C667F+^ were stained with antibodies to organelle-specific markers (Fig. 2C). ATF6 is expressed along the membrane of the ER and colocalizes with Calnexin. Further, HepG2 cells retaining the LDLR variants in the ER exhibited altered subcellular localization of ATF6 in the Golgi complex and nuclei, wherein it co-expresses with Golgin-97 and the nuclear protein Histone H3, respectively. The ER-to-Golgi transition, together with the nuclear translocation of the cytosolic domain of ATF6, which migrates to 50 kDa as seen on the immunoblot [33], demonstrates the ATF6 arm-mediated ER stress response elicited by ER-retained missense variants of LDLR. To a lesser extent, HepG2^mock^ exhibited basal expression of ATF6 within the Golgi and nuclei. As demonstrated in Fig. 2B and C, the proteolytic cleavage of ATF6 in the absence of ER stress propounds the potential importance of ATF6 in maintaining tissue homeostasis and the canonical ER stress response [34, 35]. The merged images were cropped and zoomed to 50% using imageJ software to have a clear understanding of the localization of the proteins of interest. Figure 2D represents the zoomed images of the merged panels depicted in Fig. 2C. The blue arrows represent regions where ATF6 does not colocalize with the organelle specific markers, whereas the white arrows denote regions where ATF6 colocalizes with these markers. As for GRP78/BiP, the expression of GRP78/BiP remained suspiciously low, in like manner as its transcripts, in HepG2 cells stably expressing WT or variants of LDLR and were further investigated (Fig. 2A(i) and B (iii)).

### Oxidized LDL aggravates UPR in ER stress caused by ER-retained variants

To characterize the molecular mechanisms of cellular stress response and hepatotoxicity induced by oxLDL in ER-stressed cells, HepG2^mock^, HepG2^WT+^, HepG2^D482H+^, and HepG2^C667F+^ were administered with 100 µg per ml of oxidized LDL for 24 h [36, 37]. Tunicamycin was used at a concentration of 2 µg per ml as the positive control to induce ER stress and DMSO represented the vehicle control for TM-treated cells. The dose used was selected based on previous studies employing tunicamycin on HepG2 and HeLa cells [38].

**Fig. 3:**
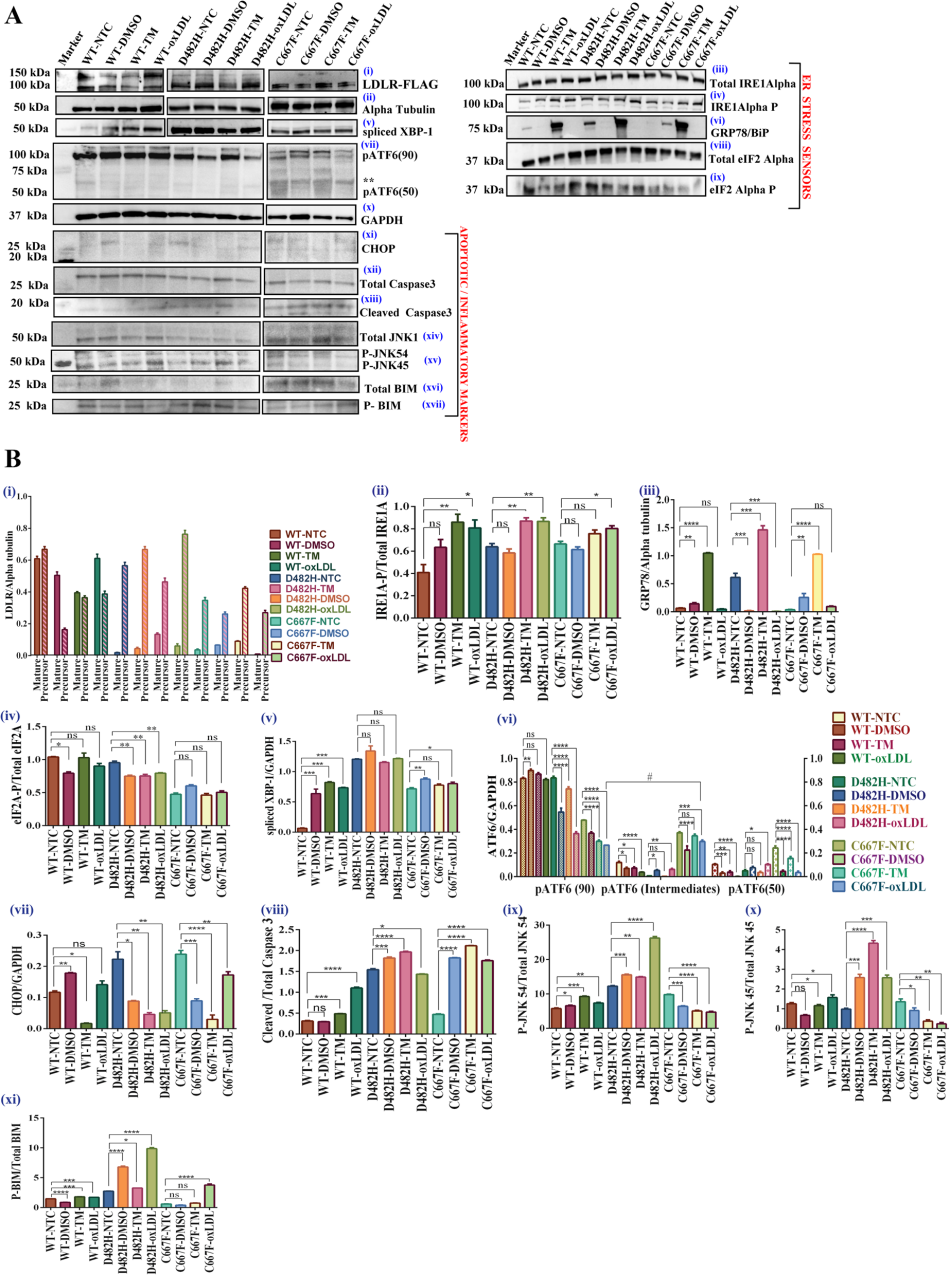
**A**-**B** Effect of oxLDL on UPR in HepG2 cells stably expressing WT /variants of LDLR **A** Western blots showing the expression pattern of ER chaperone-BiP (vi) and ER stress sensors (iii-v and vii-ix), apoptotic and inflammatory markers (xi-xvii) after 24 h of oxLDL treatment. NTC-Non treated control, TM-Tunicamycin (2 µg per ml, 24 h), DMSO-vehicle control, oxLDL-oxidized LDL (100 µg per ml, 24 h). GAPDH and Alpha tubulin were used as loading controls. Image panels were created using the QuickFigures plugin of ImageJ software, the contrast was enhanced uniformly, and confirmed that there were no significant changes with respect to proteins that exhibited minimal basal expression. ‘**’ represents the intermediate isoforms of ATF6. **B** (i-xi))-Histograms representing the change in expression of all the target proteins evaluated. For each of the four types of HepG2 cells studied (HepG2^mock^, HepG2^WT−LDLR^, HepG2^D482H−LDLR^, and HepG2^C667F−LDLR^), comparisons and statistical analyses were carried out between the respective non-treated versus treated conditions and represented as mean + SD. Statistical analyses were carried out using Dunnett’s 1-way ANOVA, or 2-way ANOVA for ATF6 (Turkey’s post hoc test), where *P* < 0.05 = *, *P* < 0. 01 = **, *P* < 0.001 = ***, *P* < 0.0001 = ****, ns = not significant, *n* = 3 replicates

Commercially available oxLDL is dissolved in PBS, and hence oxLDL treated samples were compared with the respective non-treated, non-transduced HepG2 or mock-transduced HepG2. All comparisons were carried out between the respective non-treated controls and oxLDL- treated cell types, considering the fact that the lysates for different cell types were immunoblotted on different membranes. The change in expression of target proteins that are critical players of IRE1A/XBP1-, PERK/eIF2A/ATF4/CHOP-, and ATF6- pathways were evaluated by Western blotting as represented in Fig. 3A. The corresponding histograms representing target protein expression normalized to total protein or loading control are depicted in Fig. 3B.

Tunicamycin treatment resulted in enhanced expression of all ER stress sensors and served as the positive control for ER stress induction. Compared to the respective non-treated conditions, oxLDL treatment of HepG2^WT+^ increased IRE1A-P by two folds, which remained high in oxLDL treated—HepG2^D482H+^ and HepG2^C667F+^. In the presence of oxLDL, spliced XBP-1 protein expression in HepG2^WT+^ was at par with oxLDL-treated HepG2^C667F+^ and higher in HepG2^D482H+^ **(**Figs. 3A (iii -v) and 3B (ii and iv). At the 24-h time point, we were unable to see a significant change in phosphorylated eIF2A in oxLDL- treated versus non-treated HepG2^WT+^ and HepG2^C667F+^, but a reduction was noted in HepG2^D482H+^ **(**Figs. 3A (viii and ix), 3B (iv)). An apparent depletion of pATF690 protein expression in oxLDL-treated HepG2^WT+^ was detected, with a more severe loss of the inactive ATF6 protein in oxLDL-treated HepG2^D482H+^ and HepG2^C667F+^ **(**Figs. 3A (vii) and 3B (vi). However, we noticed the abundant expression of the intermediate isoforms in HepG2^C667F+^, with a lesser but consistent expression of the nuclear ATF6 (pATF6-N) even in oxLDL treated cells, compared to the respective non-treated cells. This may be attributed to the delay in processing and trafficking of cleaved ATF6, as there was a significant increase in the levels of pATF6 intermediates than the pATF6 (90) isoforms in HepG2^C667F+^. Activation of all three branches of the UPR, as evident in ER-stressed cells expressing ER-retained LDLR variants, and to a lesser extent, in HepG2^WT+^ cells, may be attributed to the cell’s pro-survival mechanisms of maintaining cellular homeostasis. To explore the involvement of prolonged ER stress, the expression of C/EBP homologous protein (CHOP) (Fig. 3A (xi) and B (vii)), which is the central mediator of ER stress-induced apoptosis, and the apoptotic effector- cleaved caspase 3 (Fig. 3A (xii and xiii) and B (viii)) were assessed. While non-treated HepG2^WT+^, HepG2^D482H+^, and HepG2^C667F+^ displayed negligible levels of CHOP with no significant upregulation in treated conditions, exposure to oxLDL substantially increased the expression of cleaved caspase 3 in HepG2^WT+^and HepG2^C667F+^. In addition to the endonuclease activity of its activated RNase domain, the kinase domain of stimulated IRE1A propagates inflammatory and apoptotic responses along the IRE1A-TRAF2-JNK pathway. Stress-dependent signaling along the IRE1A-JNK-BIM axis extends its deleterious effects on the mitochondrial membrane by disrupting mitochondrial membrane potential. Perturbations along the MMP result in a leaky mitochondrial membrane which results in the release of the pro-apoptotic protein- Cytochrome *c* into the cytosol. This further instigates the course of apoptotic cell death and brings about mitochondrial dysfunction. To explore the cumulative effect of ER- and oxidized LDL-mediated stress in FH conditions and its likelihood of inducing inflammatory and apoptotic responses that radiate along the ER-mitochondrial interphase, the expression profiles of phosphorylated JNK and BIM were examined. In HepG2^WT+^ and HepG2^D482H+^, oxLDL treatment was capable of actuating phosphorylation of JNK-1 and JNK-2 of 45 kDa and 54 kDa, respectively. Nonetheless, the expression of these phosphorylated isoforms in oxLDL-treated HepG2^C667F+^ was lesser than in non-treated HepG2^C667F+^ (Fig. 3A (xiv and xv) and B (ix and x)). Similar to cleaved caspase 3, phosphorylated BIM expression was exalted several folds in oxLDL-treated HepG2^D482H+^, and HepG2^C667F+^, compared to the respective non-treated conditions (Figs. 3A (xvi and xvii) and 3B(xi)). Although we were unable to detect significant induction of CHOP expression in treated and non-treated HepG2^WT+^, HepG2^D482H+^, and HepG2^C667F+^, it was noted that oxLDL treatment augmented the ER stress response in cells carrying ER-retained LDLR variants. The increased levels of cleaved caspase 3 and phosphorylated BIM reveal the initiation of apoptotic signaling and progression of the UPR towards irreversible ER stress response that culminates in cell death. Enriched expression of phosphorylated JNK1 and JNK2 in HepG2^WT+^ and HepG2^D482H+^ marks the onset of inflammatory responses initiated by oxLDL in these cells. Next, we assayed the mitochondrial membrane potential (ΔΨm), cytochrome *c* release, and the extent of cytotoxicity in oxLDL treated and non-treated HepG2^mock^, HepG2^WT+^, HepG2^D482H+^, and HepG2^C667F+^. These results are elaborated in Fig. 5(A-C).

GRP78/ BiP is the master regulator of the ER stress response, wherein it precludes activation of the UPR signal transducers IRE1A, PERK, and ATF6 by remaining bound to these proteins in the normal state. Previously, a significant upregulation of GRP78/BiP along with other ER chaperones was reported in D482H- and C667F-LDLR expressing HEK293T cells than in WT-LDLR transfected cells, which confirmed the induction of ER stress by these missense variants. Despite evident changes in the UPR branches initiated by ER stress, oxLDL, or in combination, like the response in tunicamycin-treated cells, the expression of BiP was surprisingly low after 24 h of oxLDL treatment in HepG2^WT+^, HepG2^D482H+^, and HepG2^C667F^. In the respective non-treated controls, minimal levels of BiP expression were detected, with almost no expression in HepG2^C667F+^. Instead, tunicamycin treatment enhanced BiP expression in HepG2^WT+^, HepG2^D482H+^, and HepG2^C667F+^ (Fig. 3A (vi) and B (iii)).

### BiP is severely down-regulated in oxLDL treated HepG2 cells overexpressing WT- or missense variants of LDLR

**Fig. 4:**
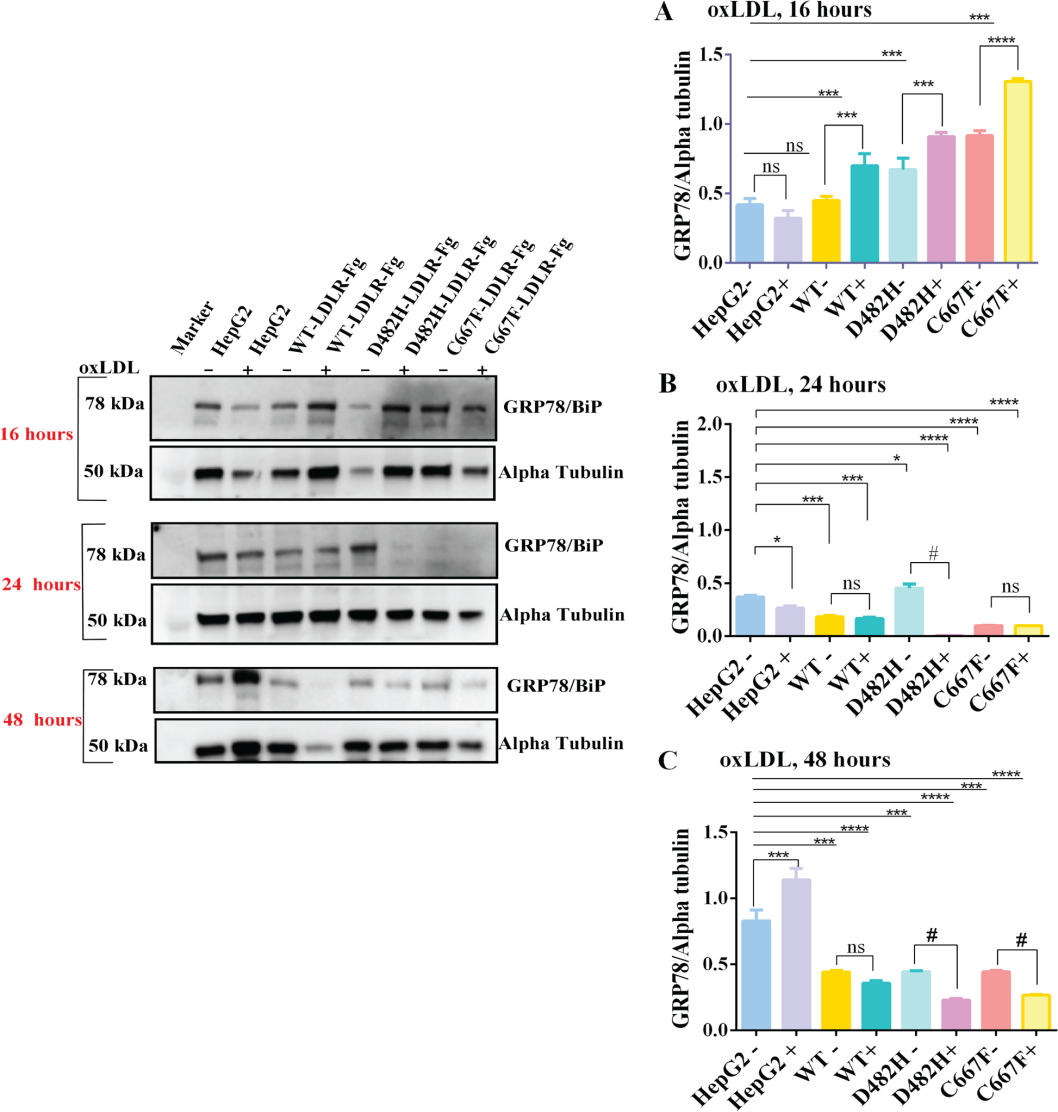
**A**-**C** BiP expression in oxLDL treated cells at various time points. Western blots depicting GRP78/BiP expression in HepG2 cells overexpressing empty vector, WT/variants of LDLR treated with 100 µg per ml of oxLDL at (**A**) 16 h, (**B**) 24 h, and (**C**) 48 h. Non- transduced HepG2 cells were used as controls to monitor the expression pattern of endogenous BiP expression at various time points. Non-treated or oxLDL-treated conditions for each cell type evaluated are marked by ‘-’ and ‘ + ’. Data are represented as mean + SD. 2-way ANOVA using Turkey’s post hoc test was used to compare BiP expression in non-treated (-) or treated ( +) conditions of each cell type with the non-treated and non-transduced HepG2 control cells (HepG2^−^). *P* < 0.05 = *, *P* < 0.01 = **, *P* < 0.001 = ***, *P* < 0.0001 = **** is represented for the comparisons between non-transduced HepG2 control cells (HepG2^−^) versus non-treated or treated HepG2^WT−LDLR^, HepG2^D482H−LDLR^, and HepG2^C667F−LDLR^. *P* < 0.05 = # when the treated cells were compared with the respective non-treated cell types. ns = not significant, *n* = 3 replicates

The interesting finding of lower expression of BiP in oxLDL treated cells at a dose of 100 µg per ml for 24 h urged us to track the expression pattern of BiP at various time points ranging from 8, 12-, 16-, 24-, and 48-h post oxLDL treatment. Figure 4 describes the expression pattern of BiP at various time points in oxLDL (100 µg per ml) treated and non-treated HepG2. Non-transduced control HepG2 (HepG2) cells were used as ‘control’ to confirm the endogenous BiP expression in oxLDL-treated (HepG2^+^) and non-treated conditions (HepG2^−^). Tunicamycin-treated cells represent the positive control for BiP induction caused by ER stress. oxidized LDL does not alter the expression of BiP in HepG2 cells at 8- and 12-h post oxLDL treatment in HepG2^WT+^, HepG2^D482H+^, and HepG2^C667F+^ (Supplemental Fig. 2(A-B)). At 16 h (Fig. 4A), oxLDL-treated HepG2^WT+^, HepG2^D482H+^, and HepG2^C667F+^ consistently expressed BiP protein, similar to the respective non-treated cells. At 24 h (Fig. 4B), although HepG2^−^, HepG2^+^, and HepG2^WT+^ expressed significant levels of BiP in non-treated and treated cells, its expression was severely downregulated in oxLDL treated HepG2^D482H+^ and HepG2^C667F+^. After 48 h of oxLDL treatment, the upregulation of BiP expression in the presence of oxLDL is an example of the classic cellular response to stress. Nevertheless, at this point, BiP expression reappeared in oxLDL-treated HepG2^D482H+^ and HepG2^C667F+^ but was intriguingly lower than in the corresponding non-treated conditions (Fig. 4C). It cannot be ruled out that overloading the hepatic cells with LDLR exerts ER stress, irrespective of being the wildtype- or the missense variants and explains the absence of BiP in oxLDL treated HepG2^WT+^.

Contrary to the pattern of upregulated targets of all three branches of UPR described in Fig. 3(A-B), the downregulation of BiP in oxLDL treated, ER stressed conditions emphasize the pathogenic involvement of oxLDL in sabotaging BiP from attending to its function as an ER chaperone and ER stress regulator, particularly in cells expressing excess LDLR. The comparatively lower expression of BiP in non-treated HepG2^C667F+^ at 24 h and its re-appearance at 48 h may be due to the delayed response of these cells to activate the ER stress signaling. While BiP maintains a low expression profile in normal cells, BiP is a bonafide cancer stem cell marker. HepG2 cells are hepatosarcoma cells, so the stress response will differ from non-cancerous cells. However, at this point, these explanations lack substantiating evidence from similar experiments conducted in non-cancerous cells.

### Overexpression of BiP in HepG2^WT+^ and ER- stressed HepG2^D482H+^/HepG2^C667F+^ restores cellular homeostasis and reduces hepatotoxicity induced by oxLDL

**Fig. 5:**
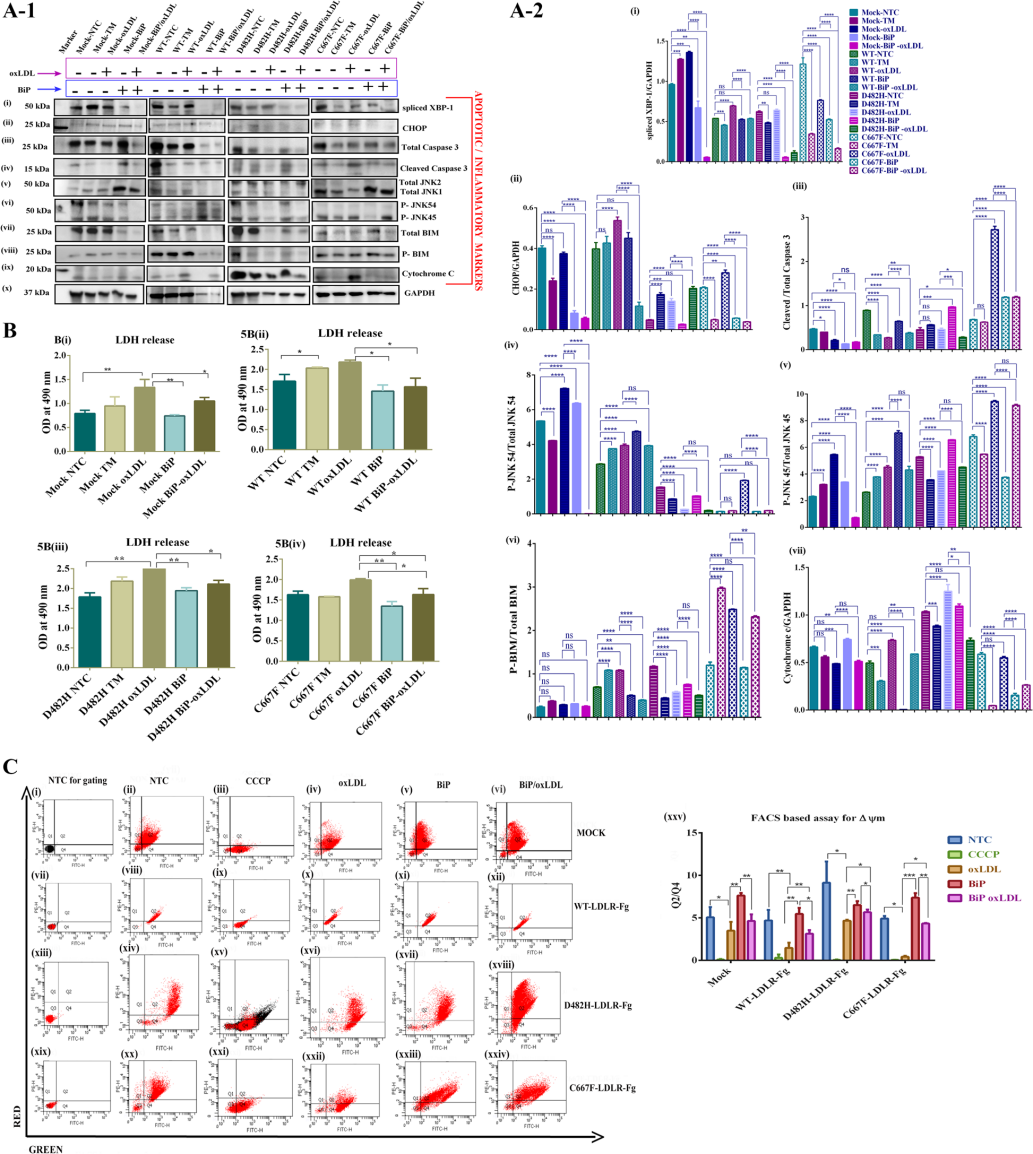
**A**-**C** GRP78/BiP mediated rescue of oxLDL and ER stress-induced hepatotoxicity. **A**-1(i-x))-Western blot panel representing the alleviation of ER stress response, apoptotic and inflammatory markers in HepG2 cells overexpressing GRP78/BiP. HepG2^mock^, HepG2^WT−LDLR^, HepG2^D482H−LDLR^, and HepG2^C667F−LDLR^ that transiently overexpress BiP are marked ‘ + ’, while cells expressing endogenous BiP are denoted with ‘-’ signs. oxLDL was administered at a dose of 100 µg per ml for 48 h and non-treated and treated conditions are denoted by ‘-’ and ‘ + ’, respectively. NTC-non treated control, TM-tunicamycin. **A**-2(i-vii))-The band intensities of GAPDH normalized target proteins were quantitated and data are represented as mean + SD. Statistical analysis was performed using One-way ANOVA (Turkey’s multiple comparison test). *P* < 0.05 = *, *P* < 0.01 = **, *P* < 0.001 = ***, *P* < 0.0001 = ****, ns = not significant. *n* = 3 replicates. **B** (i-iv))- Bar diagrams representing LDH release assay for (i) Mock (ii) WT-LDLR-Fg (iii) D482H-LDLR-Fg and (iv) C667F-LDLR-Fg-expressing HepG2 cells under various treatment conditions. Two-way ANOVA (Turkey’s post hoc test) was used to calculate statistical significance using GraphPad Prism6 software. *P* < 0.5 *, *P* < 0.001 **, *n* = 3 replicates. 5C (i-xxiv)- Representative FACS plots of non-treated control (NTC) cells used for gating (i, vii, xiii and xix); NTC as negative control (ii, xviii, xiv and xx); CCCP-treated positive controls for JC-1 assay (iii, ix, xv and xxi); oxLDL treated (iv, x, xvi and xxii); non-oxLDL treated, BiP/GRP78- overexpressing cells (v, xi, xvii and xxiii); BiP/GRP78- overexpressing, oxLDL- treated cells (vi, xii, xviii, xxiv). The histogram represents the ratio of cells exhibiting JC-1 aggregates (Q2) to monomers (Q4) as a measure of the mitochondrial membrane potential **C** (xxv)). Data represent the mean ± SD of three independent experiments. Statistical significance was determined using two-way ANOVA and Turkey’s post hoc test. *P* < 0.05, * *P* < 0.01 ** and *P* < 0.001, ***n = 3 replicates

As evident from the results described above, we observed subtle upregulation of apoptotic and inflammatory markers and severely depleted levels of BiP/GRP78 in HepG2^mock^, HepG2^WT+^, HepG2^D482H+^, and HepG2^C667F+^, treated with oxLDL at a dose of 100 µg per ml for 24 h. To understand the effect of prolonged exposure to oxLDL in HepG2 overexpressing WT or ER retained LDLR variants, we increased the duration of treatment to 48 h. This time point was chosen as we observed the reappearance of GRP78/BiP in oxLDL treated conditions 48 h post-treatment. To further investigate whether it is the absence of optimal levels of BiP that facilitates the pernicious progression of UPR to apoptosis and cytotoxicity, BiP was overexpressed in HepG2^mock^, HepG2^WT+^, HepG2^D482H+^, and HepG2^C667F+^, and then treated with 100 µg per ml of oxLDL for 48 h. Comparisons were made as described in Sect. 2.6. As evident from the Western blots for spliced XBP-1, CHOP, and cleaved caspase 3 in Fig. 5A-1 (i—iv) and A-2 (i-iii), exposure to oxLDL treatment for 48 h accentuated the activation of UPR and apoptotic pathways in HepG2^mock^, HepG2^WT+^, HepG2^D482H+^, and HepG2^C667F+^ which could be abated by the overexpression of BiP. A steep decline in spliced XBP-1 and CHOP was manifested in oxLDL treated, BiP-overexpressing HepG2^mock^, HepG2^WT+^, HepG2^D482H+^, and HepG2^C667F+^. In addition to the aforementioned apoptotic markers, P-JNK and P-BIM were compared to corroborate the involvement of inflammatory and apoptotic responses spanning the ER-mitochondrial interphase (Fig. 5A (v-viii) and A-2 (iv-vi)). Activated caspase 3 decreased by two-fold in BiP- overexpressing, oxLDL-treated HepG2^C667F+^, and a significant drop was observed in HepG2^D482H+^.

On the other hand, it remained high in HepG2^WT+^ and showed no significant change in the oxLDL- treated conditions in HepG2^mock^ cells. In all the evaluated conditions, oxLDL induced the phosphorylation of BIM, which was significantly reduced in BiP-overexpressing, oxLDL-treated conditions. HepG2^mock^ showed a dramatic decline in P-JNK 45 and P-JNK 54 in oxLDL- treated cells overexpressing BiP. In HepG2^C667F+^, 48 h of oxLDL treatment they accelerated the phosphorylation of JNK more than it had at 24 h. Besides, P-JNK45 protein outweighed the P-JNK-54 isoforms in HepG2^C667F+^ and portrayed no significant change in expression in the presence of excess BiP. At the same time, surplus amounts of BiP could bring about a two-fold reduction in P-JNK 54 in oxLDL-treated cells. HepG2^WT+^ and HepG2^D482H+^ cells did not show a significant difference in expression in the presence of auxiliary amounts of BiP at the 48-h time point. Thus, BiP helped to keep inflammation and apoptosis under control and prevented the progression toward unfavorable cellular stress and apoptosis. An evident downregulation of ER stress sensors, apoptotic and inflammatory markers as depicted in Fig. 5A-1 (i-viii) and A-2 (i-vi) reinforces the protective function of BiP in cells severely affected by oxLDL and ER stress.

The damages caused to the plasma membrane result in the release of the cytosolic enzyme lactate dehydrogenase (LDH) into the extracellular space. LDH assay was performed to compare the extent of cytotoxicity induced by oxLDL in BiP-expressing HepG2^WT+^, HepG2^D482H+^, and HepG2^C667F+^. The amount of LDH released was quantified colorimetrically and plotted as bar diagrams in Fig. 5B (i-iv). While a two-fold increase in LDLH release was displayed in oxLDL-treated HepG2^mock^ cells that express endogenous BiP, oxLDL-induced cytotoxicity was reduced by slightly over one-fold in cells overexpressing BiP. In non-treated conditions, LDH released by cells that carry an excess of BiP was lower than cells expressing endogenous BiP. A similar trend in LDH release was observed in oxLDL-treated, BiP overexpressing HepG2^WT+^, HepG2^D482H+^, and HepG2^C667F+^cells, whereby the cytotoxicity elicited by oxLDL was greatly reduced by BiP. In comparison to HepG2^mock^, LDH released by non-treated HepG2^WT+^, HepG2^D482H+^ and HepG2^C667F+^ with endogenous BiP expression was higher, suggesting the toxicity imposed on cells overexpressing LDLR. In this regard, toxicity was higher in HepG2^D482H+^ and HepG2^C667F+^ than in HepG2^WT^. Again, tunicamycin treatment induced a positive ER stress response as evident from the Western blotting and LDH assays.

Disrupted mitochondrial membrane potential (MMP) is an evident sign of mitochondrial dysfunction that simultaneously opens mitochondrial transition pores to release cytochrome *c* into the cytosol and triggers apoptotic cell death. Flow cytometric analysis of JC-1-stained cells was carried out to measure the mitochondrial membrane potential (ΔΨm) in oxLDL treated or non-treated HepG2^WT+^, HepG2^D482H+^ and HepG2^C667F+^. CCCP dye treatment disrupts the membrane potential of the cells and was used as the positive control for the assay (Fig. 5C (i-xxiv)). In healthy cells, JC-1 dye exhibits potential-dependent accumulation as aggregates in the mitochondria that emit red fluorescence at 590 nm. In apoptotic cells, the membrane potential collapses, and the JC1 aggregates dissociate into monomers as indicated by the fluorescence shift from red (~ 590 nm) to green (~ 529 nm). The ratio of red to green cells indicates the percentage of polarized (Q2) to depolarized cells (Q4) in JC-1 dye-treated cells assayed using FACS. The histogram represents the Q2/Q4 ratio in oxLDL-treated and non-treated cells that either overexpress BiP or exhibit endogenous BiP (Fig. 5C (xxv)). ER stress induced by missense LDLR variants did not collapse the MMP as evident from the Q2/Q4 ratio of ~ 4 in HepG2^mock^, HepG2^WT+^ or HepG2^C667F+^, which was two-timed higher in HepG2^D482H+^. In accordance with the findings elaborated above, the severity of disrupted MMP in HepG2^mock^, HepG2^WT+^, HepG2^D482H+^, and HepG2^C667F+^ was magnified in oxLDL treated cells, as evident by almost 50% reduction in healthy HepG2^D482H+^ and > 90% in HepG2^WT+^ and HepG2^C667F+^. The protective function of BiP is apparent in non-treated HepG2^mock^, HepG2^WT+^, HepG2^D482H+^, and HepG2^C667F+^ that overexpress BiP. A transient enhancement of BiP in oxLDL-treated cells rescued these cells from the ill effects of oxLDL and prevented disruption of MMP in HepG2^mock^, HepG2^WT+^, HepG2^D482H+^, and HepG2^C667F+^. Furthermore, oxLDL-treated HepG2^WT+^, HepG2^D482H+^ and HepG2^C667F+^ overexpressing BiP released lesser amounts of cytochrome *c* compared to the oxLDL- treated cells that endogenously express BiP (Fig. 5A-1 (ix) and A-2 (vii)). Thus, BiP imparts protective functions in non-treated HepG2^mock^, HepG2^WT+^, HepG2^D482H+^, and HepG2^C667F+^, whereas in oxLDL-treated cells, it fortifies the cell’s survival mechanisms by minimizing ER stress and hepatotoxicity.

## Discussion

Hypercholesterolemia, per se, is marked by surfeit levels of LDL in circulation that are highly susceptible to oxidative modification. These oxidized LDL moieties are highly toxic to the cell as they elicit oxidative stress responses within the cell. By and large, it is the cardiovascular system that is primarily affected and hence, extensively studied [39, 40]. In patients with FH caused by p.D482H and p.C667F substitutions in LDLR, the lack of expression of the membrane-bound, mature LDLR induces ER stress. Further, these ER-retained variants induce ER stress response as evidenced by the elevated levels of spliced X-box protein (XBP-1 s) transcripts and are degraded by the HRD-1/SEL1 mediated ERAD machinery [31]. Physiologically, the UPR of the ER is essential for maintaining cellular homeostasis in hepatocytes during lipid metabolism and protein secretion. Hepatic diseases associated with dyslipidemic conditions lead to over-accumulation of fat deposits in hepatocytes- as in steatosis, which is reversible. However, steatosis coupled with inflammatory responses that terminate in apoptosis gives rise to steatohepatitis and eventually progresses toward hepatocarcinoma [41]. Data from pharmacologic ER stress induction in genetic knockout mouse models of UPR components identified hepatic steatosis as a common occurrence in livers with sustained, excessive, or unresolved ER stress [42–44]. Considering the significance of lipid metabolism in liver homeostasis, we investigated the molecular nexus between oxLDL and ER stress to unveil the severity and course of damage imparted to hepatic cells.

The ER stress response machinery is a conserved signaling system that communicates adaptive or maladaptive stimuli by activation of all three foundational genes of the ER stress sensor [45]. Fascinatingly, in a zebrafish model of fatty liver demonstrated by Cinaroglu et al., while ATF6 prevents hepatic steatosis induced by acute ER stress, it potentiates steatosis in chronic ER stress [46]. In this study, abnormally elevated IRE1 and PERK kinases, and proteolytically cleaved transcription factor ATF6 in HepG2 cells retaining the missense variants of LDLR were identified. Despite the increase in transcripts of the proapoptotic marker- CHOP, the protein expression of CHOP and activated caspase 3 were relatively stable in WT- and variants of LDLR, suggesting the activation of the primary ER stress response to pro-survival mechanisms under ER stress induced by ER retained variants. P58IPK is an ER-stress-induced molecular chaperone that tightly regulates protein synthesis and folding in the ER by delaying the phosphorylation of eIF2A [47]. Likewise, EDEM1 targets terminally misfolded proteins for ERAD-mediated degradation [48]. A steady increase in *EDEM1* together with a dip in *P58IPK*—the inhibitor of PERK kinase was noted, which provide evidence in favor of the elicited UPR.

Accumulating evidence for the detrimental effects of FH on lipid metabolism has identified oxLDL induced oxidative stress as the key player in causing mitochondrial injury and endothelial cell dysfunction [49–51]. Studies based on experimental models and human subjects identified oxLDL as the pathogenic determinant of Non-Alcoholic Fatty Liver Disease (NAFLD)-related diseases [52, 53]. In the murine model for Non-Alcoholic Steatohepatitis (NASH) established by Yimin et al., short-term administration of oxLDL in the high fat diet fed mice exacerbated hepatic steatosis and gave rise to inflammatory responses as manifested in NASH [54]. Apart from oxLDL, dysregulated ER stress unequivocally progresses towards maladaptive UPR and promotes hepatocellular damage and inflammation as in steatosis and steatohepatitis [55, 56]. It is therefore reasonable to assume the existence of a crosstalk between ER stress and oxLDL in FH that upsets homeostasis in the liver, eventually causing hepatic injury [57]. In support of this hypothesis, it was observed that within 48 h of exposure to oxLDL, CHOP expression and activation of caspase 3 were enhanced, particularly in ER-stressed HepG2 expressing the missense variants of LDLR. The changes observed in the expression of apoptotic and inflammatory markers in oxLDL treated cells at the 24-h time point were subtle, and eventually became distinct by 48 h. Again, cells expressing WT- and variants of LDLR did not exhibit precisely similar changes in expression along all three branches of ER stress sensors and downstream targets, as each cell type differ in their response to stress, and the time taken for transcriptional, translational or post translational processing of these targets. When the intrinsic adaptive mechanisms fail to re-establish homeostasis, hepatocytes capitulate to these external or internal pressures by adopting apoptotic or necrotic cascades [58–60]. One mechanism by which CHOP induces apoptosis is by the induction of BIM- the BCL-2 inhibitor [61, 62]. Studies on liver biopsies of NASH patients, and murine or cellular models [42–44] exhibit profuse amounts of proinflammatory cytokines and apoptotic cascades, which provide compelling evidence for inter-organelle communication between ER-originating UPR and the mitochondria [63, 64]. In line with these findings, here the data illustrate that increased cytotoxicity imposed by oxLDL in ER stressed FH conditions propagate inflammatory responses and apoptotic signals to the extent of mitochondrial dysfunction by disrupting the mitochondrial membrane potential.

However, as with any ER stress response, as illustrated by the large proportion of data available, it was expected to see the enhanced expression of GRP78/BiP transcripts and protein in HepG2 expressing the ER-retained LDLR variants, both in the presence or absence of oxLDL. On the contrary, despite the explicit upregulation of ER sensors that interact with BiP via their luminal domains, these cells failed to express BiP when treated with oxLDL at a dose of 100 µg per ml for 24 h. This prompted the authors to chase the expression of BiP in HepG2 at various time points to gain more insight into the link between BiP and ER- or oxLDL-induced stress in the hepatic scenario. The re-appearance of trivial levels of BiP protein after 48 h of oxLDL treatment in ER-stressed HepG2 intrigued us to analyze whether topical expression of BiP was capable of relieving hepatic ER stress and duly rescuing the cells from being predestined to death. From the data described in this paper, it is evident that the cytotoxic responses elicited by oxLDL originated within the ER and spread along to the mitochondria, where it disrupted the MMP. A disturbed potential gave rise to leaky mitochondria, as manifested by elevated cytochrome c within the cytosol. Ectopically increasing BiP expression in cells treated with oxLDL was capable of restoring the hampered MMP and lowering the cytochrome c release to the cytosol. Taken together, BiP extends its protective function to multiple organelles and holds a critical place in hepatoprotective mechanisms that need to be extensively delved into in other in vitro model systems.

GRP78/BiP is a member of the heat-shock protein 70 (HSP70) superfamily that resides in the ER lumen [65]. Besides being established as an ER-resident molecular chaperone, BiP is also identified as an ER sensor that directly activates UPR [66–68]. Adding on, mouse NASH tissues and palmitate-treated hepatoma cells portrayed increased expression of BiP [69–71]. In contrast, data obtained from studies on hepatic tissues of NASH patients discovered the dysregulated profile of BiP as evident from the absence of BiP in these cases [72]. Earlier, depletion of GRP94 in mouse liver was found to increase LDL-c and promoted tumorigenesis in mouse hepatic progenitor cells [73, 74]. BiP overexpression in the liver of ob/ob mice reduces hepatic steatosis and partially protects HepG2 from palmitate-induced ER stress and apoptosis by attenuating the induction of CHOP [75, 76]. The Liver-specific Grp78 Knock Out (LGKO) mouse model demonstrated by Ji et al. [77] manifested hepatic apoptosis, inflammation, and ER dilation, suggesting the importance of adequate levels of Grp78 in maintaining ER homeostasis in the liver [78]. In line with the depleted GRP78 in liver tissues of NASH patients discussed above, the data provide substantial in vitro evidence for the pivotal role played by GRP78/BiP in maintaining liver homeostasis.

GRP78 is not confined to the ER but is also present in other cellular compartments such as the mitochondria, nucleus, cytoplasm or plasma membrane. The accumulation of misfolded proteins in the ER lumen and lipid disequilibrium induces the activation of UPR sensors by the ER and mitochondrial resident chaperones that regulate protein quality control and metabolic balance.

In addition to its chaperone function in regulating protein folding, GRP78 plays an inevitable role in maintaining ATP and calcium homeostasis owing to its calcium and ATP binding properties [79]. By doing so, it plays an arbitrary role in keeping apoptosis under check and sustains intricate inter-organelle balance between the ER and mitochondria [80]. p58IPK functions as a co-chaperone and regulator of GRP78 in the ER lumen by stimulating ATP hydrolysis and recruiting GRP78 to the site of misfolded proteins. Conclusive evidence for the protective role of overexpressed GRP78 in protecting astrocytes from ischemic injury along the ER-mitochondrial axis and in stabilizing calcium concentration substantiates the findings reported here [81].

This report highlights the pathogenic relevance caused by the downregulation of p58IPK and BIP in derailing the inter organelle crosstalk by destabilizing calcium homeostasis, disrupting the mitochondrial membrane potential and imparting apoptotic and inflammatory responses.

### What is known and what is new?

Most of the evidence identifies GRP78/BiP as the gold-standard marker protein that is upregulated under conditions of ER stress. In addition to what is discussed in the above sections, this paper reports the oscillating expression of BiP, evident by its mysterious disappearance at the 24 h-time point and the reappearance within 48 h of ER stress induction provides exciting and interesting leads to investigate BiP dynamics in the liver. Sparing one study on the absence of BiP in NASH patients, knowledge on the role of BiP and its regulatory functions in the hepatic system is still in its infancy.

### Study strength and limitation

The authors ensured that the in vitro cellular system used for the study truly represented ER-stressed hypercholesterolemic conditions by using cells that stably overexpressed either WT- or variants of LDLR. This was achieved by lentiviral transduction of HepG2 cells. Although transient transfection is a rapid and easy method of introducing genes of interest into cells, the efficiency of transient transfection of HepG2 with WT/variants of LDLR was as low as < 10%. The high success rates of generating stable and robust lines of hard to transfect cells and the larger amounts of DNA (> 9 kb) that can be transferred with ease into cells make lentiviral transduction a popular technique for genetic manipulation. However, lenti particle-mediated transduction is laborious, time-consuming and poses more risks than chemical-based transfection methods. In addition, the viral titer needs to be as high as possible to ensure that the cells are infected with highly efficient viral particles. Moreover, the efficiency of viral particles drastically drops by almost 50% if repeatedly freeze-thawed. This is overcome by snap-freezing single-use of lentiparticles, of approximately 10^^7^ transfection units (TU) per ml. The seeding density of cells, purity and yield of the plasmids used for infecting the cells, multiplicity of infection (MOI) of viral particles, duration and dose of the antibiotics chosen for selecting positive clones and are other critical factors that need to be optimized while using lentiviral transduction. In this study, these factors were critically taken care of and a transduction efficiency of > 95% of positive cells was achieved.

The liver is a vital metabolic organ that functions as the kingpin in lipid and protein homeostasis. The vast majority of data on disrupted lipid homeostasis, wherein the interplay between oxLDL and ER stress comorbidities associated with FH are extensively studied, primarily focusing on the cardiovascular system, insulin resistance, and obesity. To the best of the authors’ knowledge, this is the first report on the combined effect of ER stress induced by missense variants of LDLR that are pathogenically associated with familial hypercholesterolemia and oxLDL as contributing factors to diseases. Although widely accepted as a suitable hepatic cell line to study hepatotoxicity and the convenience of culturing HepG2, the major limitation of this study is the use of these hepatosarcoma cells as the model system. The transcriptome and proteome profile of HepG2 may not be a true representation of normal hepatocytes owing to its cancerous properties. An alternative to using this model system would be to use primary human hepatocytes (PHH) isolated from liver. However, PHH are difficult to maintain as the reproducibility is questionable due to the lack of viability of the cells in culture [82, 83]. The experiments were cautiously designed and tightly controlled with ample replicates to avoid ambiguities during data interpretation. The strength of this report is that it gives substantial evidence in line with similar findings from various other groups that have used HepG2 cells as the cellular model to tease out the factors contributing to hepatic ER stress caused by saturated fatty acid-induced apoptosis [84, 85].

The overexpression of GRP78 on tumor cells—particularly on the cell surface of glioblastomas, lung, and liver cancers, than on normal cells, makes it a promising therapeutic target and imaging biomarker for cancer [86]. In any case, future studies are warranted to determine whether the proposed mechanism of BiP-mediated rescue is recapitulated in normal hepatocytes, human iPSC-derived hepatocytes, or bio-printed 3D models of normal liver tissues.

## Conclusion

**Fig. 6:**
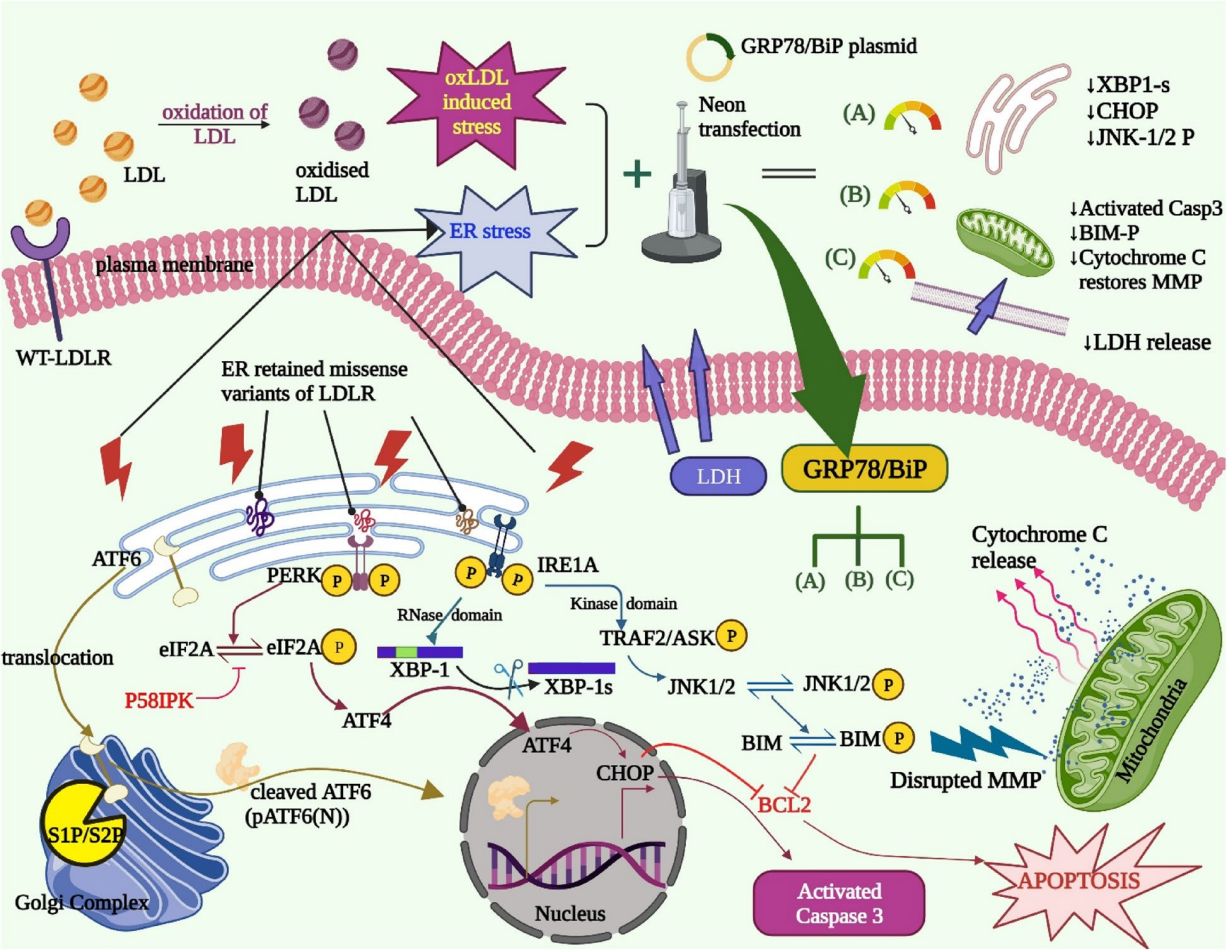
A graphical abstract summarizing the study model and major findings. The thin black arrows and red lightning symbols represent ER stress-caused by ER retention of LDLR, and oxLDL induced stress- caused by oxidation of excess levels of LDL. The brown, maroon and blue colored arrows represent ER stress dissipated along the ATF6, PERK and IRE1A branches, respectively. The pacman symbol denotes S1P/S2P protease in the Golgi Complex. The red inhibitory arrows denote the proteins that inhibit/are inhibited during the process. The scissors symbolize splicing of XBP-1 transcripts. The blue dots and red arrows in the mitochondria denote release of cytochrome C caused by disrupted mitochondrial membrane potential. Purple arrows-direction of LDH release from cells due to cytotoxicity. The continuous low-medium dial denotes restored cellular responses brought about by neon transfection of GRP78/BiP plasmid along (**A**) ER, (**B**) mitochondria and (**C**) plasma membrane. The downward arrows denote lowering of target proteins evaluated

The authors report the haphazard ER stress response in HepG2 cells overexpressing either WT- or variants of LDLR that exhibit downregulated expression of BiP, at the transcript and protein levels, and its implications in the progression of the most common liver diseases. The prolonged accumulation of WT or variants of LDLR in hepatocytes induces ER stress and delays the clearance of circulating LDL from the system, thereby increasing the susceptibility to be modified to toxic derivatives such as oxLDL. ER stress coupled with oxLDL-induced stress act as a double-edged sword that derails cellular homeostasis and causes hepatic injury. In this study, we elucidate the protective function of BiP in hepatic cells severely affected by the deadly duo- oxLDL and ER stress, wherein BiP safeguards the cells by reducing cytotoxicity, restoring homeostasis, and preventing the spread of damage along the ER-mitochondria axis. Figure 6 illustrates the summary of the major findings illustrated as a graphical abstract. In further support of this conclusion, overexpressing GRP78 and GRP94 in ER-stressed HepG2 protected the cells from ER-stress-induced cell death and is proven to carry out anti-apoptotic functions [87]. The data provide interesting leads that identify familial hypercholesterolemic patients as highly susceptible to developing hepatic insults with molecular signatures similar to those manifested in NAFLD and NASH, thus implicate its clinical relevance in disease prognosis and diagnosis. These findings open new arenas that are critically relevant to the clinicians in providing better prognostic and diagnostic tools, by identifying BiP or other ER stress molecules along the signaling pathway, as therapeutic targets and adopting measures that alleviate apoptotic responses, prevent or slow down the pathogenic progression of reversible NAFLD to irreversible NASH in patients with FH.

## Supplementary Information

**Additional file 1: Figure. 1.** Sanger sequencing to confirm site-directed mutagenesis of FLAG-tagged WT-LDLR. **Figure. 2.** (A-B)-BiP expression in oxLDL treated cells at early time points.

## Data Availability

“The dataset(s) supporting the conclusions of this article is (are) included within the article (and its additional file(s)).”

## Abbreviations

ORF: Open reading frame
DDK: FLAG (Fg) epitope
ΔΨm: Mitochondrial membrane potential, rpm-revolutions per minute
PI: Propidium Iodide
mRNA: Messenger ribonucleic acid
