# Supplementary material for: GRP78/BiP alleviates oxLDL-induced hepatotoxicity in familial hypercholesterolemia caused by missense variants of LDLR in a HepG2 cellular model
Source: Lipids Health Dis. 2023 May 29;22:69. doi: 10.1186/s12944-023-01835-x (PMC10226256; doi:10.1186/s12944-023-01835-x)
Supplement: Supplementary file 1 — Additional file 1: Figure. 1. Sanger sequencing to confirm site-directed mutagenesis of FLAG-tagged WT-LDLR. Figure. 2. (A-B)-BiP expression in oxLDL treated cells at early time points. [file 12944_2023_1835_MOESM1_ESM.pdf]

Supplemental Figure 1

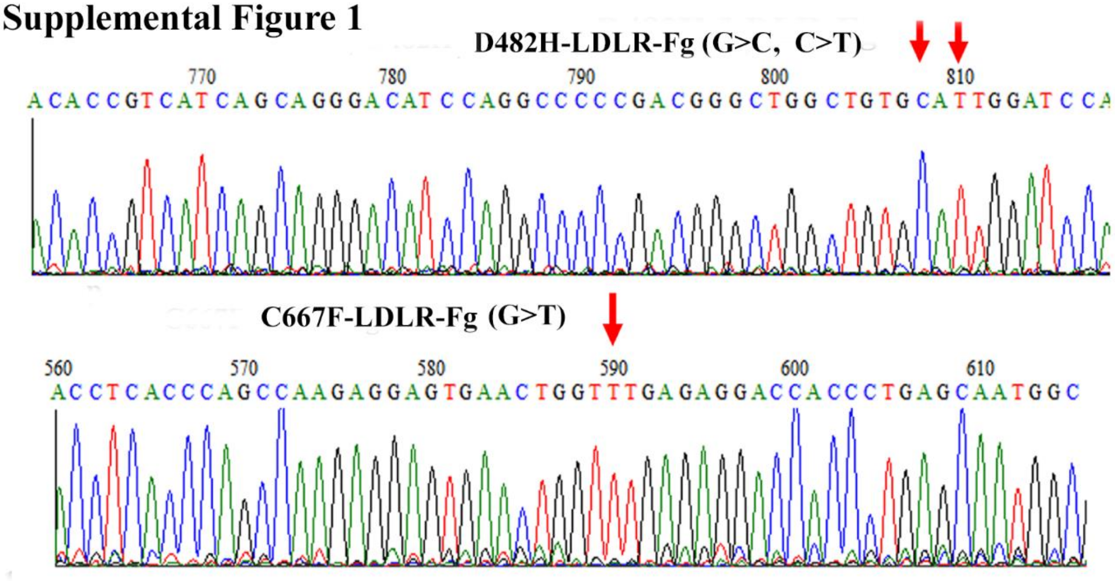

Supplemental Figure 1- Sanger sequencing to confirm site-directed mutagenesis of FLAG-tagged WT-LDLR. The red arrows on the chromatograms indicate the location of the nucleotides substituted to generate the open reading frames of the ER-retained, missense variants of LDLR- p.D482H-LDLR (top) and p.C667F-LDLR (bottom).

Sup.Figure 2

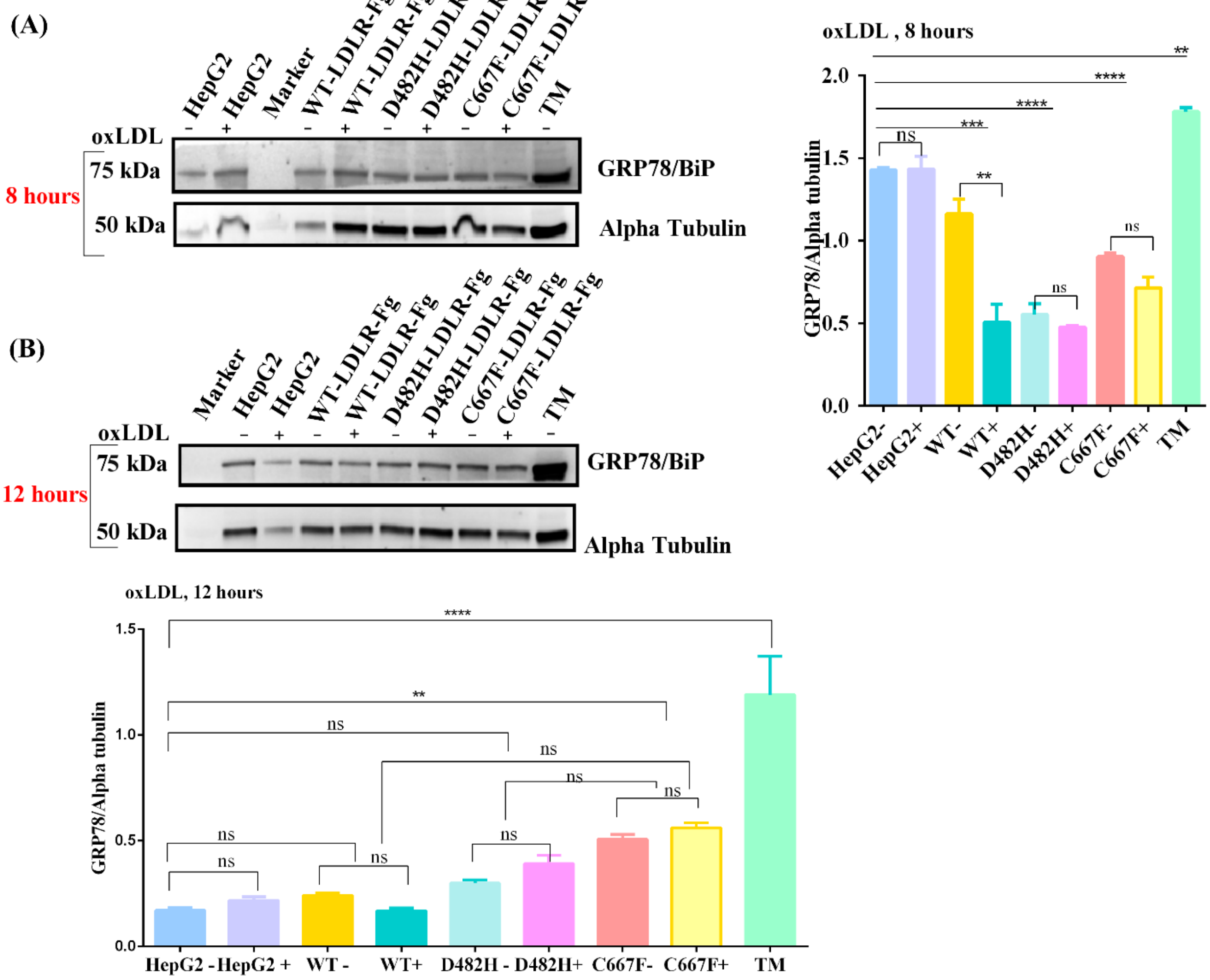

Supplemental Figure 2(A-B)-BiP expression in oxLDL treated cells at early time points. Western blots depicting GRP78/BiP expression in HepG2 cells overexpressing empty vector, WT/variants of LDLR treated with 100 µg per ml of oxLDL at (1A) 8 hours and (1B) 12 hours. Bar diagrams denoting Alpha-tubulin normalized BiP expression at these time points are represented as mean +SD. 2-way ANOVA using Turkey's post hoc test was used to compare the corresponding treated (+) *versus* non-treated (-) conditions for each HepG2 cell type, wherein p<0.05=* or #, p<0.01=**, p<0.001=***, p<0.0001=****, is represented for the comparisons between non-transduced HepG2 control cells (HepG2-) versus non-treated or treated $HepG2^{WT\text{-}LDLR}$, $HepG2^{D482H\text{-}LDLR}$, and $HepG2^{C667F\text{-}LDLR}$. P<0.05 =# when the treated cells were compared with the respective non-treated cell types. ns=not significant, n=3 replicates.
